# Exploratory General-Response Cognitive Diagnostic Models with Higher-Order Structures

**DOI:** 10.1017/psy.2025.15

**Published:** 2025-04-16

**Authors:** Jia Liu, Seunghyun Lee, Yuqi Gu

**Affiliations:** Department of Statistics, Columbia University, New York, NY, USA

**Keywords:** Cognitive Diagnostic Models, identifiability, Monte Carlo Expectation–Maximization (MCEM) algorithm, probit model, Q-matrix

## Abstract

Cognitive Diagnostic Models (CDMs) are popular discrete latent variable models in educational and psychological measurement. While existing CDMs mainly focus on binary or categorical responses, there is a growing need to extend them to model a wider range of response types, including but not limited to continuous and count-valued responses. Meanwhile, incorporating higher-order latent structures has become crucial for gaining deeper insights into cognitive processes. We propose a general modeling framework for higher-order CDMs for rich types of responses. Our framework features a highly flexible data layer that is adaptive to various response types and measurement models for CDMs. Importantly, we address a challenging exploratory estimation scenario where the item-attribute relationship, specified by the Q-matrix, is unknown and needs to be estimated along with other parameters. In the higher-order layer, we employ a probit-link with continuous latent traits to model the binary latent attributes, highlighting its benefits in terms of identifiability and computational efficiency. Theoretically, we propose transparent identifiability conditions for the exploratory setting. Computationally, we develop an efficient Monte Carlo Expectation–Maximization algorithm, which incorporates an efficient direct sampling scheme and requires significantly reduced simulated samples. Extensive simulation studies and a real data example demonstrate the effectiveness of our methodology.

## 1 Introduction

Cognitive Diagnostic Models (CDMs), or Diagnostic Classification Models (Templin & Henson, 2010), have emerged as a crucial tool for modeling educational assessment data with multidimensional discrete (often binary) latent variables. Various diagnostic goals lead to CDMs with different measurement models; examples include the Deterministic Input Noisy Output (DINA; “And” gate model Junker & Sijtsma, 2001) with the conjunctive assumption, the Deterministic Input Noisy Output “Or” gate model (DINO; Templin & Henson, 2006) with the disjunctive assumption, the main-effect diagnostic models (de la Torre, 2011; DiBello et al., 2012; Maris, 1999) incorporating the additive effects of latent attributes, and the all-effect general diagnostic models (de la Torre, 2011; Henson et al., 2009; Von Davier, 2008) with a more saturated parameterization.

Currently, most existing CDMs are designed to model binary or polytomous response data. However, the diversification of examination modes and increased availability of educational and psychological data have enabled the collection of various response data types. Continuous response data arise in many scenarios, such as language proficiency tests scoring on a continuous scale and recording response time in computer-based assessments (Minchen et al., 2017). The modeling of response times has long been a topic of interest, see De Boeck & Jeon (2019) for a comprehensive overview. Another common response type is count responses, found in assessments recording the number of correct responses, the frequency of specific behaviors in classroom activities, the usage frequency of particular strategies in problem-solving tasks, and computer-based tests recording visit counts per item (Liu et al., 2022; Man & Harring, 2019). Rasch (1993) first proposed a Poisson-based item response theory (IRT) model for count data, and since then, many other models have been developed (Magnus & Thissen, 2017; Man & Harring, 2019, 2023).

A crucial element in a CDM is the relationship between observed item responses and latent attributes, specified by the Q-matrix (Tatsuoka, 1983). Recently, Lee & Gu (2024) proposed a new cognitive diagnostic modeling framework for general response types with a prespecified Q-matrix. However, in many practical applications, the true Q-matrix may not be known a priori, necessitating an exploratory approach to infer the Q-matrix directly from the response data. In such challenging exploratory settings for flexible data types, estimating the Q-matrix reliably and efficiently is highly desirable but largely unknown. Beyond exploratory CDMs and general response types, integrating a higher-order layer into CDMs (de la Torre & Douglas, 2004; Templin et al., 2008) offers significant advantages. Such models uses one or more continuous latent traits to explain the binary attributes, providing a more nuanced understanding of the relationships between different skills. This yields a comprehensive and realistic representation of cognitive processes.

This article makes the following key contributions. *First*, we propose a unified framework for modeling higher-order general-response CDMs (HO-GRCDMs). We formulate the bottom layer (data layer) of HO-GRCDMs using flexible exponential family distributions. This allows the model to directly adapt to (a) different types of responses (binary, continuous, count, etc.) by altering the parametric family and (b) various types of measurement assumptions (main-effect, all-effect, DINA, etc.) by modifying the latent covariate vector. In the higher-order layer, we employ a probit model to describe the relationship between the higher-order continuous latent traits and the binary latent attributes. We consider both pre-specified and unknown higher-order structures, referring to the former as the *partially exploratory* setting and the latter as the *fully exploratory* setting. The higher-order modeling approach was originally proposed by de la Torre & Douglas (2004) for binary response data, referred to as the higher-order CDM. We generalize it to general response types and employ a probit link instead of the logit link used in de la Torre & Douglas (2004). As will be discussed later, using a probit link for the higher-order layer provides significant theoretical and computational advantages.

*Second*, we establish identifiability for the proposed HO-GRCDMs. Model identifiability is a crucial prerequisite for valid statistical estimation, but it is a challenging issue for complex latent variable models such as HO-GRCDMs. While the identifiability of single-layer exploratory CDMs for categorical data has been extensively studied (e.g., Chen et al., 2020; Culpepper, 2019; Liu & Culpepper, 2024; Xu & Shang, 2018), much less is known about the identifiability of CDMs with higher-order structures. Lee & Gu (2024) provides identifiability results for general response CDMs with a known *Q*-matrix, but that also does not guarantee the identifiability of an HO-GRCDM. Some existing studies established identifiability for CDMs with higher-order *discrete* latent structures, including the Bayesian pyramid model in Gu & Dunson (2023), and the DeepCDM in Gu (2024). However, the identifiability issue of CDMs with higher-order continuous latent traits is still underexplored, despite these models’ popularity (Culpepper & Balamuta, 2023; de la Torre & Douglas, 2004; Templin et al., 2008; Wang, Yang, et al., 2018). Culpepper & Balamuta (2023) addresses the identifiability of the CDM layer in a higher-order model, but does not establish identifiability of the entire model. To our best knowledge, our identifiability results are the first to fully identify CDMs with multidimensional higher-order continuous latent traits.

*Third*, we propose an efficient Monte Carlo Expectation–Maximization (MCEM) algorithm for estimating HO-GRCDMs. The computational challenge of HO-GRCDM arise from three aspects: (a) the various types of response data, (b) the complex hierarchical structure that consists of both binary and continuous latent variables, and (c) and the unknown Q-matrix. A typical approach to parameter estimation of such models is to regard the latent variables as missing data and employ Markov chain Monte Carlo (MCMC; Robert & Casella, 2004) or an EM-type algorithm, where the latter method is usually faster. However, the complex hierarchical structure in our model makes the maximization of the complete data log-likelihood intractable during the typical EM updates. In this article, we propose an MCEM algorithm to maximize the regularized maximum likelihood to simultaneously estimate the Q-matrix and other parameters. Similar to Chen et al. (2015), we consider the Q-matrix estimation as a latent variable selection problem, and maximize log-likelihood with 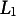 penalty. Our method provides the first non-MCMC method for parameter estimation in the category of CDMs with a multidimensional higher-order structure.

Our estimation framework incorporates an efficient direct sampling scheme and features significantly reduced simulated samples. The continuous latent traits 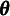 in the higher-order layer are the only latent variables whose realization need to be sampled. After imputing the missing data 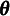 , the M-step optimization becomes more tractable, and we solve this by the cyclical coordinate ascent (Friedman et al., 2010; Tay et al., 2023). Here, the simulation of 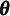 has been a crucial issue in both IRT and higher-order CDM estimation. The MCMC method, commonly used for this purpose, often suffers from slow convergence and requires careful tuning of algorithm parameters. In this article, we highlight that, benefiting from the use of a probit link for the higher-order layer, we can directly simulate 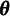 from a unified skew-normal distribution according to the recent theoretical results of Li et al. (2025). Last but not least, initialization is an important issue for the efficiency of an algorithm. We employ an efficient Singular Value Decomposition (SVD)-based method for finding initial values, which is an extension of Chen et al. (2019) and Zhang et al. (2020) from the binary response case to the general response case. This non-iterative method is computationally fast and enjoys statistical consistency guarantees under certain conditions (Zhang et al., 2020).

The rest of this article is organized as follows. Section 2 introduces the framework of HO-GRCDM. Section 3 presents the identifiability results. Section 4 proposes an efficient MCEM algorithm for parameter estimation. Sections 3 and 4 are developed under a partially exploratory setting (with an unknown *Q*-matrix in the CDM layer), while Section 5 extends the methodology to a fully exploratory setting (with an unknown *Q*-matrix in the CDM layer and also an unknown higher-order 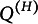 -matrix in the deeper layer). Section 6 conducts extensive simulation studies for HO-GRCDM under various measurement models, higher-order structures, and response types. Section 7 applies our methodology to a response time data set extracted from the TIMSS 2019 math assessment. Section 8 concludes and discusses future research directions. The Supplementary Material contains additional theoretical results, all technical proofs, and additional numerical results.

## 2 Model setup

Assume there are *N* examinees responding to a test with *J* items. For each examinee, the observed response vector 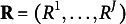 is a *J*-dimensional vector. Depending on the assessment design, the response 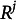 could be binary, polytomous, counted-valued, continuous, and so on. We represent an examinee’s latent skill profile as a *K*-dimensional random vector, 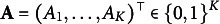 , and let 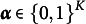 be an arbitrary binary vector. Let 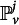 denote the prespecified parametric family for the *j*th response, with parameter 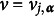 . Before introducing the specific notations, we first present the general form of HO-GRCDM as, 
(1)

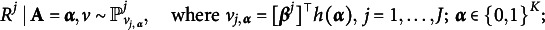



(2)

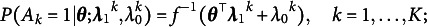



(3)

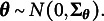


Equations (1)–(3) describe a CDM with a three-layer hierarchical structure, with the observed general-response data 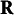 at the bottom layer, the binary latent attributes 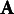 at the middle layer, and the higher-order Normal latent variables 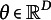 at the top layer.

A CDM typically consists of two main components: the measurement part and the latent part. The measurement part, defined in (1), describes how the observed responses 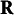 depend on the latent attributes 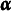 . We consider a very broad framework that allows flexible response types and various measurement models. The latent part, defined in (2) and (3), models the binary attributes. In the following sections, we separately define each part of the HO-GRCDM. For notational simplicity, for a positive integer *M*, let 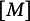 be the set of all positive integers 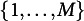 .

### 2.1 Bottom data layer: CDMs with general responses (GR-CDMs)

In the bottom layer (1), an examinee’s observed responses depend on his/her statuses of *K* binary latent attributes. Here 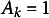 indicates the *k*th attribute is mastered; otherwise, 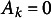 . The 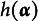 is a latent covariate vector consisting of certain main effects and interaction effects of attributes, and 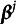 is a parameter vector that we will further specify. The parameter 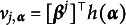 is a linear combination of 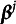 and 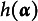 . For convenience of presentation, we assume that for all items 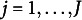 , the 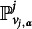 are the same parametric family, and omit the superscript.

We first elaborate on the choice of the parametric family 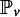 that can be used to model each response type. Below, we denote 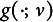 as the probability mass/density function (pmf/pdf) of the parametric family under consideration. The Bernoulli distribution with mean 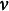 can be used to model binary responses (de la Torre, 2011; Maris, 1999). Alternatively, one can assume that 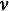 is the logit transform of the Bernoulli mean, as for the case of the Logistic Linear Model (LLM, Maris, 1999). Equivalently, we model the Bernoulli mean as 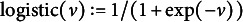 : 
(4)

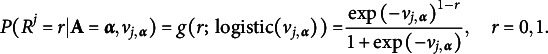

In this work, we allow very diverse choices of non-categorical responses such as count and continuous data. For count-valued data, the Poisson distribution with mean 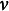 can be used: 
(5)

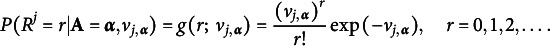

For unbounded continuous responses, one can use a normal distribution, where 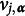 denotes the mean parameter. Together with an additional variance parameter 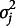 , we have 
(6)

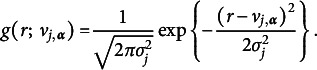

For continuous responses with a constrained range, one can use transformed-normal distributions. For example, the log-normal distribution can be used for modeling positive responses: 
(7)

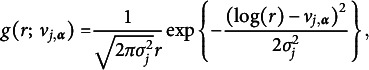

and the logistic-normal distribution for responses within the range of (0,1): 
(8)

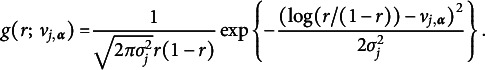

Alternatively, to model positive continuous responses, we can also use the Gamma distribution: 
(9)

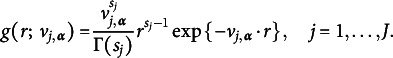

Here, 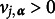 is the rate parameter, and 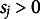 is the shape parameter.

Next, we specify the assumptions on the parameter 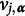 . In the literature of cognitive diagnostic modeling, a common assumption involves a pre-specified *Q*-matrix (Tatsuoka, 1983), 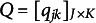 , that describes which of the *K* attributes are measured by each of the *J* items. If 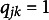 , then the *k*th attribute is measured by the *j*th item; otherwise, 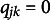 . So, the value of 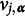 must depend only on the attributes specified by the *j*th row of the *Q*-matrix. While the *Q*-matrix is often assumed to be provided along the data, we consider a more challenging *exploratory estimation* scenario where the *Q* is unknown and need to be estimated along with other parameters.

Given the *Q*-matrix, one needs some structural assumptions on how 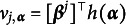 depends on the *Q*-matrix entries. Here, we present three popular measurement model assumptions on the parameters 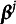 and the function 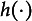 . First, the *main-effect GR-CDM* assumes that 
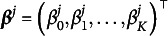
 , and 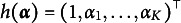 . Then, we can write Eq. (1) as 
(10)

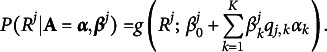

For illustration, suppose that we are considering binary responses and 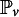 is the Bernoulli distribution, then (10) becomes the Additive Cognitive Diagnosis Model (ACDM; de la Torre).

Next, the *all-effect GR-CDM* assumes 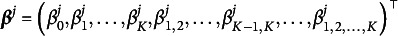 , and 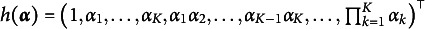 . The pmf/pdf becomes 
(11)

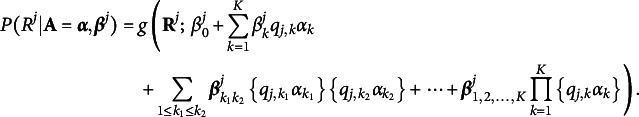

Note that for the case of binary responses, this becomes the GDINA model (de la Torre, 2011).

Finally, we introduce the GR-DINA model. We borrow the notations from the all-effect models, and take 
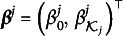
 and 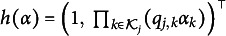 . Here, 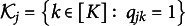 denotes the set of attributes that are measured by item *j*. Then the pmf/pdf is written as 
(12)

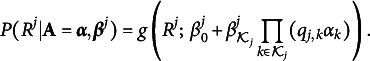

Note that the above formulation can be regarded as a special case of all-effect GR-CDM. Under binary responses, this model is the popular DINA (Junker & Sijtsma, 2001) model with the conjunctive assumption. Under positive continuous responses, (12) becomes the continuous DINA model (c-DINA; Minchen et al., 2017).

For main-effect and all-effect GR-CDMs, the *Q*-matrix should constrain certain 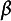 -coefficients to be zero. For example, under the main-effect model, we must have 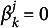 for *k* for which 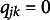 . Under the all-effect model, we must have 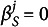 when 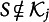 . Since we consider an unknown *Q*-matrix, the index of such zero coefficients is also unknown and needs to be estimated from data.

### 2.2 Latent layers: Higher-order latent trait model for binary attributes

As shown in Equations (2) and (3), we consider a higher-order latent layer to model the attributes 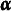 . We introduce a *D*-dimensional *continuous* latent trait, 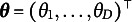 . There are two common choices for the invertible link function *f*: the logit link function 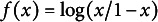 , and the probit link function 
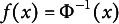
 , where 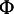 is the cumulative distribution function (CDF) of a standard normal random variable. In this article, we employ the probit link function and let 
(13)

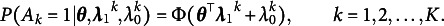

Here, 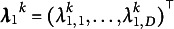 and 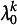 are the slopes and the intercept, respectively. Let 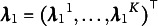 be a matrix consisting of slope parameters of all *K* attributes, and 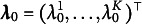 be a vector consisting of intercept parameters of all *K* attributes. We assume a pre-specified binary matrix 
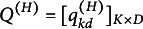
 , which constrains the sparsity structure of 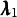 to enhance the interpretability. The entry 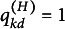 implies that the *d*th continuous latent variable 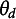 contributes to the mastering of the *k*th binary latent attribute 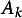 . We refer to the HO-GRCDM with an unknown *Q*-matrix and a pre-specified 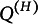 matrix as a *partially exploratory* setting, and refer to the case where both *Q* and 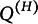 are unknown as a *fully exploratory* setting. In the following sections, we first develop the identifiability theory and estimation algorithm under the partially exploratory setting, and then extend the methodology to the fully exploratory case in Section 5. In Equation (3), 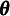 is assumed to follow a *D*-variate normal distribution, 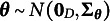 , where the zero mean vector 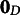 is to fix the measurement origin, and the covariance matrix 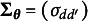 has unit diagonal entries to fix the measurement units. That is, 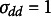 , for 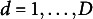 . We also assume that the first item loading on each factor is positive to resolve the sign indeterminacy issue of the latent factors.

The higher-order latent layer is introduced to resemble an item response model (Birnbaum, 1968; Lord, 1952). This modeling approach was initially used in de la Torre & Douglas (2004), where the authors considered binary responses and assumed both the bottom layer’s *Q* matrix and the latent layer’s 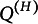 matrix were known. In addition to the higher-order CDM, another approach introduced by Templin et al. (2008) employed a multivariate probit model with a *single* continuous latent factor. In that work, each binary attribute is derived by dichotomizing a Normal random variable at a specific threshold, with the *K* Normal variables modeled using a factor analysis structure. Going beyond binary responses, Culpepper & Balamuta (2023) proposed a CDM for polytomous responses that similarly model the higher-order latent structure via a probit model with a single latent factor.

Intuitively, the higher-order latent structure should be more parsimonious than the bottom layer, as it generally represents more abstract factors/traits in a higher level. For this aim, a *subscale structure* for 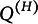 may be appropriate, where each attribute is associated with only one latent trait among all the *D* traits. In other words, an attribute 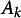 exclusively depends on a latent trait 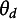 , and we have 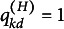 . For all other groups indexed by 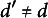 , we assume 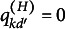 and 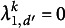 to not include their effect on 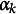 . However, in some cases, this structure may be too simple to capture the relationship between 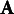 and 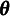 . To address this, the *bifactor structure* with an additional general factor could be a more flexible alternative yet still being parsimonious. In the bifactor structure, we assume that there are 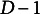 groups (indexed by 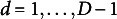 ), and that each binary attribute is assigned to exactly one group. Each attribute 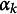 that belongs in group *d* is influenced by two latent factors: a general factor 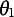 , and a group-specific factor 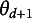 . Consequently, we have 
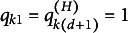
 . The effects of the other groups 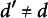 are assumed to be zero, that is 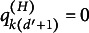 and 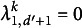 . In the remainder of the article, we will assume that the higher-order model follows either the subscale structure or the bifactor structure.

### 2.3 Benefits of using the probit link to model the higher-order layer

We elaborate on our rationale for choosing the probit link to model the higher-order layer, as opposed to the more common logit link. There are mainly two advantages in doing so. To see this, we first present the marginal distribution of the binary random vector 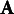 obtained using the probit link. For each 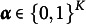 , the marginal probability of 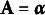 under our model is 
(14)

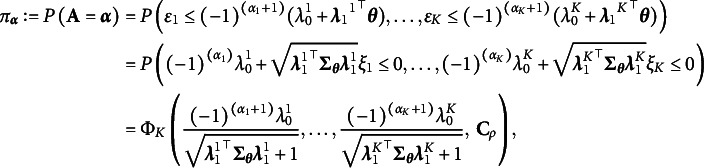

with a tetrachoric correlation matrix 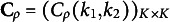 : 

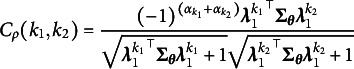

for 
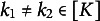
 , and 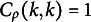 for 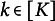 . Here, 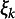 and 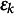 are independent and identically distributed standard normal random variables, and 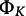 represents the CDF of a *K*-variate normal distribution; see more details in Fang et al. (2021).

The first virtue of using the probit link is for establishing model identifiability. Using (14), Fang et al. (2021) proved that the probit model’s identifiability boils down to identifying the parameters 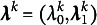 based on the threshold values 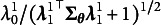 , and the pairwise tetrachoric correlations. This means that identifiability is reduced to a problem similar to the identifiability of linear factor models. In contrast, this property does not exist when using a logit link, which involves a complex convolution of Gaussian and logistic random variables.

The second advantage of using a probit link is on computation. First, the explicit form of the marginal distribution of 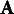 in (14) significantly reduces the computational complexity of parameter estimation. When using an EM-type algorithm, once the conditional expectation 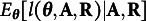 , where 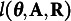 is the log-likelihood, is computed, the whole conditional expectation 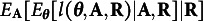 in the E-step is available, as the out-layer expectation can be easily computed according to Equation (14). Second, utilizing the probit link enables the development of an efficient sampling scheme, as it allows direct sampling of 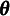 from the target distribution 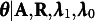 . As detailed in Section 4.2.1, 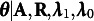 follows a unified skew-normal distribution, whose samples can be obtained by a direct combination of samples from truncated normal and multivariate normal distributions.

## 3 Identifiability

We next propose conditions that guarantee the identifiability of HO-GRCDMs. For a statistical model 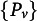 indexed by a set of parameters 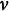 , we say that the model is identifiable at the true parameter 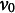 when the equality between marginal distribution of the observed variables 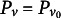 implies equal parameter values 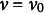 . Identifiability is a fundamental prerequisite for consistent parameter estimation and valid model interpretation.

We first present separate identifiability conditions for (a) the bottom layer *exploratory* GR-CDM and (b) the higher-order continuous latent layer *as if the binary attributes were observed binary responses*. Then, we combine these results using a layer-wise proof argument similar to that in Gu (2024) and derive identifiability conditions for HO-GRCDMs. More specifically, we first marginalize out the top continuous layer and identify the GR-CDM parameters, including the proportion parameters 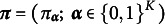 describing the marginal distribution of the binary attributes. Next, we use the estimated GR-CDM proportion parameters 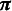 and (14) to identify the parameters in the higher-order probit model. We define saturated GR-CDMs as follows.

Definition 1 **(Saturated GR-CDM).**

A saturated GR-CDM with parameters 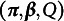 is a CDM without an higher-order structure, defined by (1) and with proportion parameters 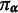 for each binary attribute pattern 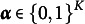 . Here, 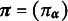 satisfy 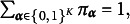 and 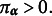

We impose a monotonicity condition on the item parameters to avoid the sign-flipping for each latent attribute; that is, to distinguish between 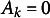 and 1. Motivated by the popular monotonicity conditions for binary-response CDMs (Xu & Shang, 2018), we assume that 
(15)

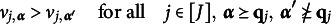

holds for all saturated GR-CDMs, and also for all HO-GRCDMs. Here, 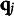 is the *j*th row of *Q*. The assumption (15) means that the students possessing all required skills for the *j*th item have a larger 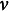 -parameter in the distribution 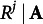 than those who lack some required skills. This condition can be further simplified under specific GR-CDMs. For example, (15) is equivalent to 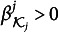 under the DINA model, and to 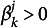 for 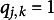 under the ACDM.

Proposition 1. Under the saturated DINA/main-effect/all-effect GR-CDM that satisfies the monotonicity condition (15), the model components 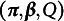 are identifiable up to a permutation of the *K* latent attributes when the following conditions hold.

- The true *Q*-matrix contains two submatrices 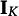 after row swapping, i.e., *Q* can be written as 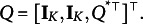

- Suppose that the *Q*-matrix is written as in A. For any 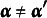 , there exists 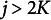 such that 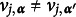 .

In particular, condition B holds when the *Q*-matrix also contains another identity submatrix 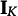 .

Note that Proposition 1 is a very general result stated under any GR-CDM not necessarily with a higher-order structure. Conditions A and B resemble popular identifiability conditions for CDMs with categorical responses (Culpepper, 2019; Xu & Shang, 2018). Lee & Gu (2024) showed that these conditions also suffice for identifying GR-CDMs, but under the confirmatory setting with a known *Q*-matrix. Proposition 1 above further ensures that if the unknown *Q*-matrix satisfies these conditions, then the *Q*-matrix itself and the model parameters are both uniquely identifiable.

Next, we present identifiability conditions for the higher-order probit layer in HO-GRCDMs. Here, we view the probit model as a parametric family with parameters 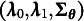 and probability mass function in (14). Recall that we consider the subscale model and the bifactor model with a known higher-order loading structure 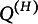 to model the *K* binary attributes.

We first present the necessary and sufficient conditions for the subscale model, which is proved by heavily utilizing the properties of the probit link mentioned after (14). This identifiability result may be of independent interest in the IRT literature. We present the proofs of all theoretical results in the Supplementary Material.

Proposition 2. Consider the subscale model with *K* attributes and *D*-dimensional Gaussian latent factors. For 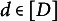 , let 
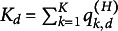
 denote the number of attributes that belong to group *d*. Then, the model is identifiable if and only if one of the below conditions holds for all 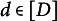 :

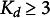
 ,

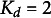
 , 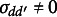 for some 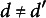 .

To summarize the above conditions C1 and C2, the subscale model with three or more attributes for each group is identifiable. Interestingly, to ensure identifiability, we require at least two attributes to belong to each group, which boils down to assuming condition A on the 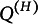 -matrix.

Next, we present identifiability conditions for the bifactor model, where the *D*-dimensional latent vector 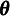 consists of one general factor 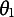 and 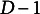 group-specific effects 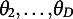 . Here, it is necessary to assume an additional orthogonal structure between the general and group-specific factors to resolve a trivial rotational ambiguity issue (see Fang et al., 2021, Section 4.1). For each 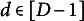 , let 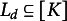 be the collection of attributes that belong to the *d*th group. We also let 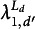 be a sub-vector of 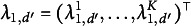 that consists of the indices 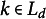 .

Proposition 3 **(Fang et al. (2021, Theorem 7)).**

Consider the bifactor model with *K* binary attributes and *D*-dimensional Gaussian latent variables with covariance 
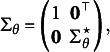
 where 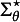 is a 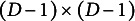 symmetric positive definite matrix with 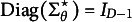 . Let 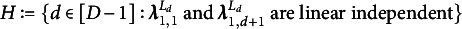 . Then, the model is identifiable if 
(16)

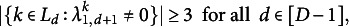

and one of the following conditions hold 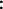

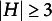
 ,

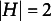
 , and there exists a group *d* such that 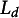 can be partitioned into 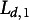 and 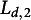 so that 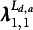 and 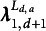 are linearly independent for both partitions 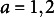 .

Combining the above results, we can establish the desired identifiability result for HO-GRCDMs. The main argument is to sequentially identify the latent layers, similar to previous works for multilayer variants of CDMs (Gu, 2024; Gu & Dunson, 2023).

To resolve the latent attribute permutation issue in Proposition 1, we additionally assume that there is a pre-specified *anchor item* for each latent attribute. This means for each latent attribute 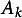 , we know that some item 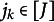 solely measures 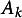 . Without loss of generality, combined with condition A, we can assume that the first *K* items are the anchor items for the *K* binary attributes.

Theorem 1. The following two identifiability conclusions hold.

- Consider an HO-GRCDM with a **subscale** higher-order layer and known anchor items. The model is identifiable when the true bottom layer GRCDM parameters 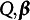 satisfy conditions A and B, and the true latent layer parameters 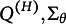 satisfy either condition C1 or C2.
- Next, consider an HO-GRCDM with a **bifactor** higher-order layer and known anchor items. The model parameters are identifiable when the true parameters satisfy conditions A, B, (16), and either C3 or C4.

Theorem 1 provides our main result that guarantees the HO-GRCDMs are identifiable. Even with the anchor item assumption, we are still identifying all other 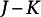 rows of the *Q*-matrix.

One may notice that the knowledge of anchor items is usually not required for identifying exploratory CDMs without a higher-order structure. However, this anchor assumption is required for our unique partially exploratory setting where we want to utilize the available higher-order 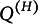 -matrix. Without anchor items, the *Q*-matrix in the bottom CDM layer is only identifiable *up to a label permutation of the K attributes, just like in typical one-layer CDMs with a fully unknown Q-matrix*; in this case, the rows of the 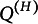 -matrix cannot be aligned with the columns of the *Q*-matrix. To relax this anchor-item requirement, we also consider a fully exploratory setting in Section 5, where both *Q* and 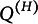 are treated as unknown.

## 4 A new MCEM algorithm

In this section, we develop an MCEM algorithm to estimate HO-GRCDMs. We first define some notations. Suppose there are *N* subjects responding to a test with *J* items. Let 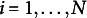 and 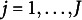 denote the index of subjects and items, respectively. The test is designed to measure *K* binary latent attributes for each subject *i*, 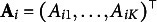 , which is further determined by *D* higher-order continuous latent variables, 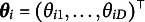 . We slightly abuse notation and use 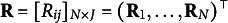 to denote the observed response matrix. Let 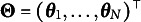 be the 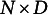 matrix that collects continuous latent variables for the *N* subjects, and define 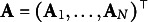 as the 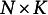 matrix consisting of the binary attribute profiles for all *N* subjects.

Let 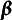 and 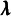 denote the set of all the coefficient parameters in the first (1) and second layer (2), respectively. Additionally, we aim to estimate the *Q*-matrix in the CDM layer and the covariance matrix 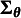 for the continuous latent traits (3). Note that estimating *Q* by directly maximize the marginalized log-likelihood is computationally infeasible even for a moderate size of *J* and *K*. This is because one need to compute the profile likelihood based on each *Q* among 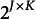 possible matrices, and find out the one that maximizes the profile likelihood. One solution to avoid such expensive computation is to consider the estimation of *Q* as a latent variable selection problem and solving it through a regularized maximum likelihood estimator (Chen et al., 2015). In particular, we maximize the regularized marginal log-likelihood 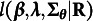 with an 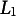 penalty 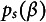 : 
(17)

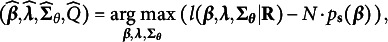

where the log-likelihood function can be written as 
(18)

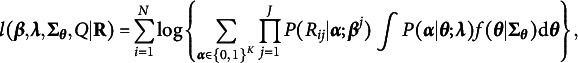

and the penalty function is defined as 
(19)

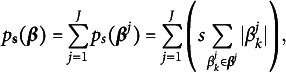

where *s* is the regularization parameter. Here, the *Q*-matrix does not need to appear explicitly in the log-likelihood expression because its information is implicitly captured by the sparsity of the coefficients. After solving (17), 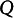 can be estimated by identifying the non-zero pattern of 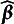 .

The mechanism for identifying the entries 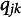 in the *Q*-matrix varies across different measurement models. For *main-effect* models, 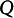 can be recovered using the rule 
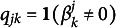
 , where 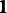 is the indicator function. For *all-effect* models, theoretically, each row of 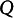 should be identified by the highest-order non-zero coefficient. Specifically, if 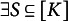 such that 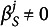 and 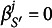 for all 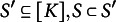 , then 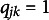 for 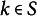 ; otherwise, 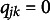 . However, this strict rule may not be always applicable because some estimated 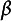 -coefficients may be close to zero but not exactly zero. In practice, a more effective approach is either to choose the largest non-zero interaction coefficient or to truncate the coefficients before identifying 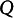 . For the latter approach, we recommend practitioners set the truncation thresholds based on the general magnitude of their estimated coefficients. For the *DINA model*, since there should be only one non-zero coefficient for each item 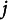 , the largest non-zero interaction coefficient can be selected, and the corresponding 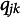 can be identified as equal to one.

The maximization problem presented in Equation (17) is quite complex due to the summation of integrals inside the log function and cannot be solved directly. The Expectation–Maximization (EM) algorithm is a popular method that iterates between the E-step and the M-step to seek the maximizer, and we first introduce the penalized variant of the EM algorithm in Section 4.1. This basic EM algorithm still suffers from the intractable integrals in the E-step, which motivates us to propose a more scalable novel MCEM algorithm in Section 4.2.

### 4.1 Penalized EM algorithm

We first introduce the basic procedure of a penalized EM algorithm. A usual EM algorithm alternates between two steps: in the E-step, the expected complete data log-likelihood is computed, and in the M-step, the parameters are updated by maximizing this expected log-likelihood. The penalized EM algorithm follows the same procedure but includes a penalty term in the M-step to regularize the parameter estimates.

The complete data log-likelihood in a HO-GRCDM is 
(20)

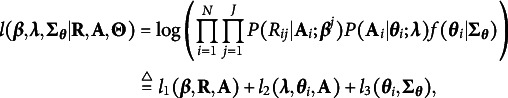

with 
(21)

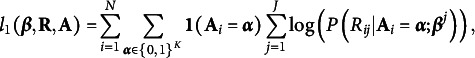



(22)

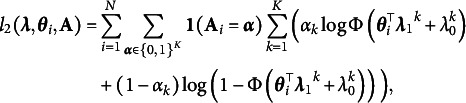



(23)

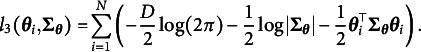



Let 
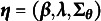
 generically denotes the collection of all parameters. For any parameter, a superscript “ 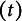 ” denotes the values obtained in the *t*th iteration. Each iteration *t* of the penalized EM algorithm contains the following two steps:

**E-Step**: Compute the *Q*-function as the expectation of the complete data log-likelihood:
(24)

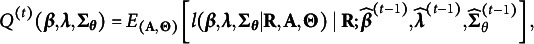

where the conditional expectation is with respect to 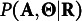 . We break down 
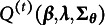
 into three parts, 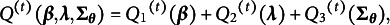 with 
(25)

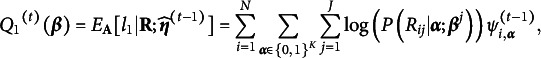

 Here the common term 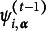 has the following expression, 
(28)

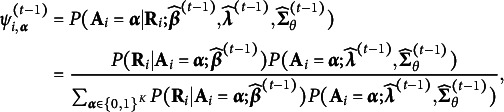

and 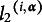 and 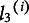 denote the corresponding terms in the summation indexed by *i* and 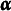 presented in Equations (22) and (23), respectively.

**M-Step**: Update the parameters by maximizing the penalized *Q*-function: 
(29)

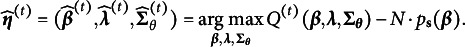

This is typically a convex optimization for exponential family distributed responses.

### 4.2 MCEM algorithm with an efficient sampling scheme

#### 4.2.1 Monte Carlo integration for E-Step

As mentioned earlier, the probit link offers a significant advantage by enabling direct computation of the conditional expectation 
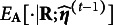
 as shown in (25)–(27), due to the explicit form of 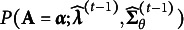 presented in (14). This simplifies the computational challenge of the E-Step to calculating the inner expectations, 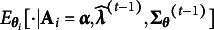 , as involved in (26) and (27). Furthermore, conditional on 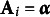 , 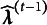 , and 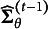 , 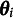 ’s are independent and identically distributed (i.i.d.). Using a general notation 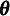 to represent 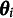 in Equations (26) and (27), we have 
(30)

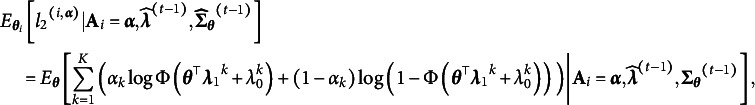

and 
(31)

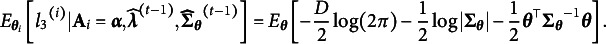

This implies when computing 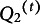 or 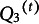 , only 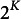 expectations, 
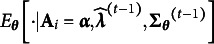
 , for 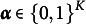 , need to be evaluated, regardless of the sample size *N*.

Despite the significantly reduced computational complexity, the above expectations involve multidimensional integrals and cannot be evaluated in closed form. We propose to use Monte Carlo integration and draw 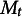 samples 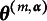 , 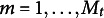 from 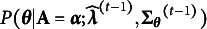 , 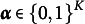 , in the *t*th iteration. For any function 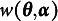 of 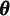 and 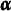 , we can approximate its expectation as 
(32)

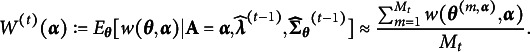

By replacing 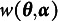 with the corresponding terms within the square brackets in Equations (30) and (31), we obtain Monte Carlo approximations for these expectations.

Sampling from 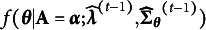 has been a challenging issue in the literature of both multidimensional IRT models and higher-order CDMs. A commonly used method is the MCMC method, including the Metropolis–Hastings (MH; Cai, 2010) sampler and the Gibbs sampler (Béguin & Glas, 2001; Culpepper, 2016). However, such methods suffer from slow convergence to the target distribution, thereby slowing down the algorithm. Fortunately, with a probit link used in the latent layers, directly sampling is feasible. By Li et al. (2025, Theorem 4.2), 
(33)

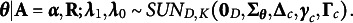

Here, 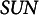 denotes an unified skew-normal distribution (Arellano-Valle & Azzalini, 2006), where 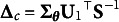 , 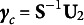 , 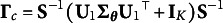 , and 
(34)

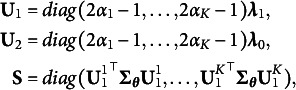

with 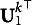 denoting the *k*th row of 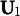 . Furthermore, by Li et al. (2025, Corollary 4.3), if (33) holds, then the conditional distribution of 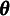 is equal to the following distribution (where “ 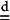 ” means “equal in distribution”) 
(35)

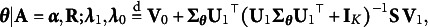

where 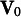 is independent of 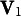 and 
(36)

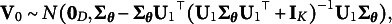



(37)

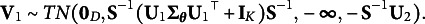

Here, 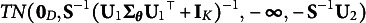 denotes a K-variate truncated normal distribution with zero mean and covariance matrix 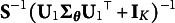 and truncation below the threshold 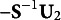 . This means that we can generate samples of 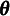 by first drawing samples of 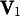 and 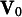 , and then performing a linear combination of these. The sampling scheme is summarized in Algorithm 1. This approach is efficient, as sampling from both the multivariate normal distribution and the truncated normal distribution is straightforward.

#### 4.2.2 The M-Step implementation

In M-Step, the maximization of 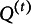 can be divided into three optimization problems. 
(38)

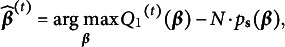



**Figure:**
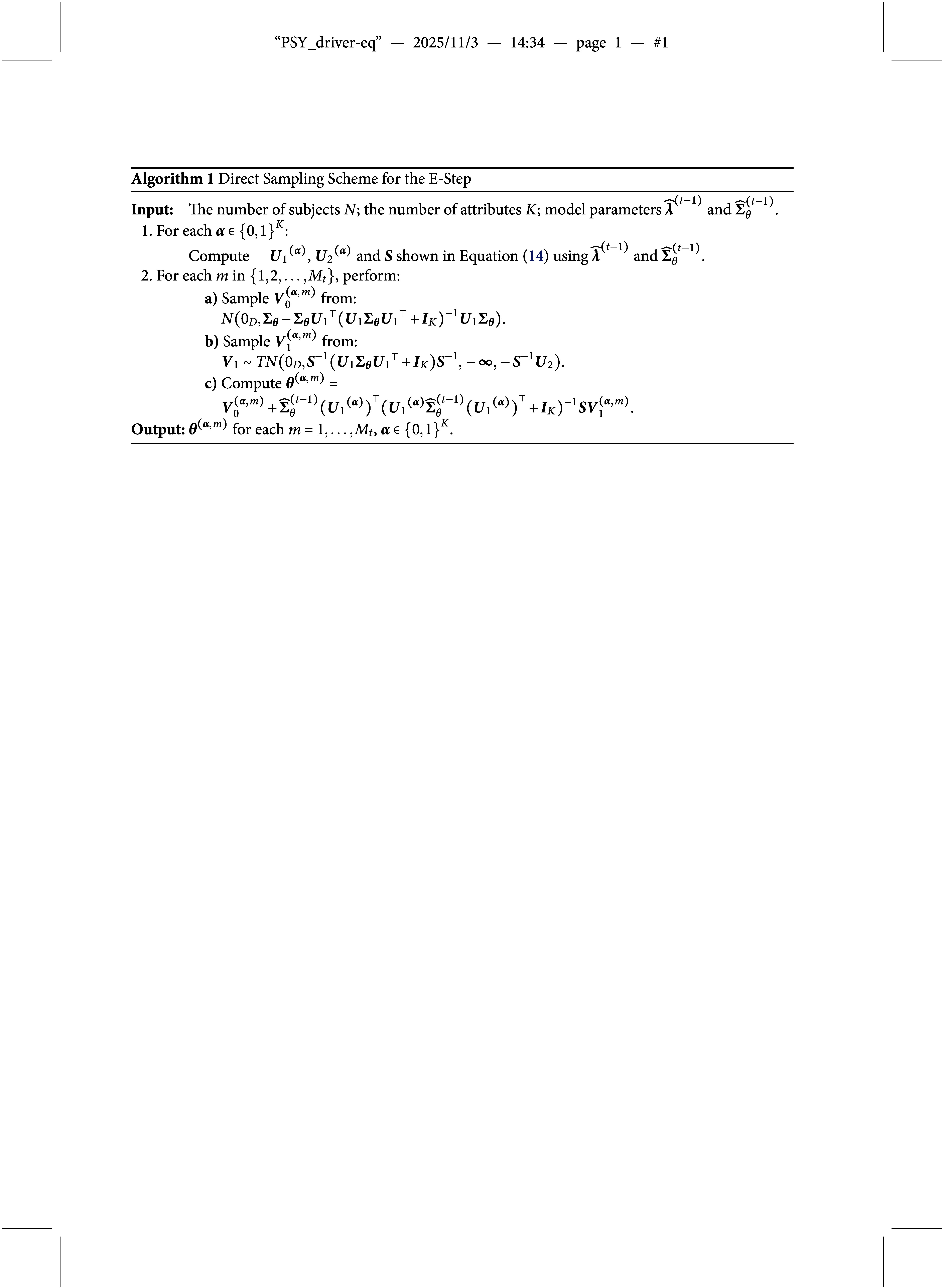



(40)

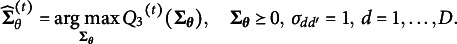

The optimization objective in (38) incorporates an 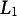 penalty and utilizes various functions 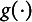 according to the response data type and measurement models. The optimization of (39) falls within the field of generalized linear models with a probit link. We use the coordinate ascent method, as described in Friedman et al. (2010) and Tay et al. (2023), to solve both problems. This method is known for its flexibility and power in solving such optimization problems.

Regarding the optimization of Equation (40), to estimate a covariance matrix with the constraint that diagonal elements must be ones, we employ a method inspired by the approach used in Stan (Carpenter et al., 2017) and detailed by Lewandowski et al. (2009). Below, we elaborate on this method as a two-step estimation procedure. First, we estimate 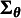 without considering the constraints, and denote the resulting estimator as 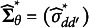 . In each iteration *t*, according to Equations (27) and (31), this solution can be directly derived as, 
(41)

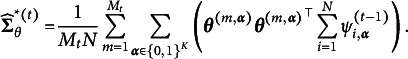

For simplicity, we neglect the iteration-specific notation 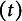 and introduce the computation based on a general 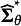 .

Next, we compute the final estimator 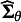 that satisfies our diagonal constraints by reparameterizing the variance by its Cholesky decomposition 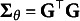 . Let 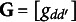 , and let 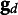 denote the *d*th column of 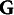 . To ensure the diagonal entries of 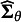 are ones, the upper triangular Cholesky factor 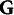 must satisfy 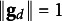 . Based on 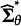 , we can use the following procedure to obtain 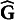 : 
(42)

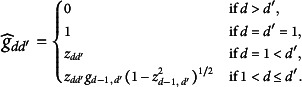

Here, 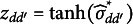 . After obtaining 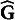 , we compute 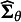 based on 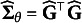 .

We summarize the above MCEM algorithm for the HO-GRCDM in Algorithm 2.

**Figure:**
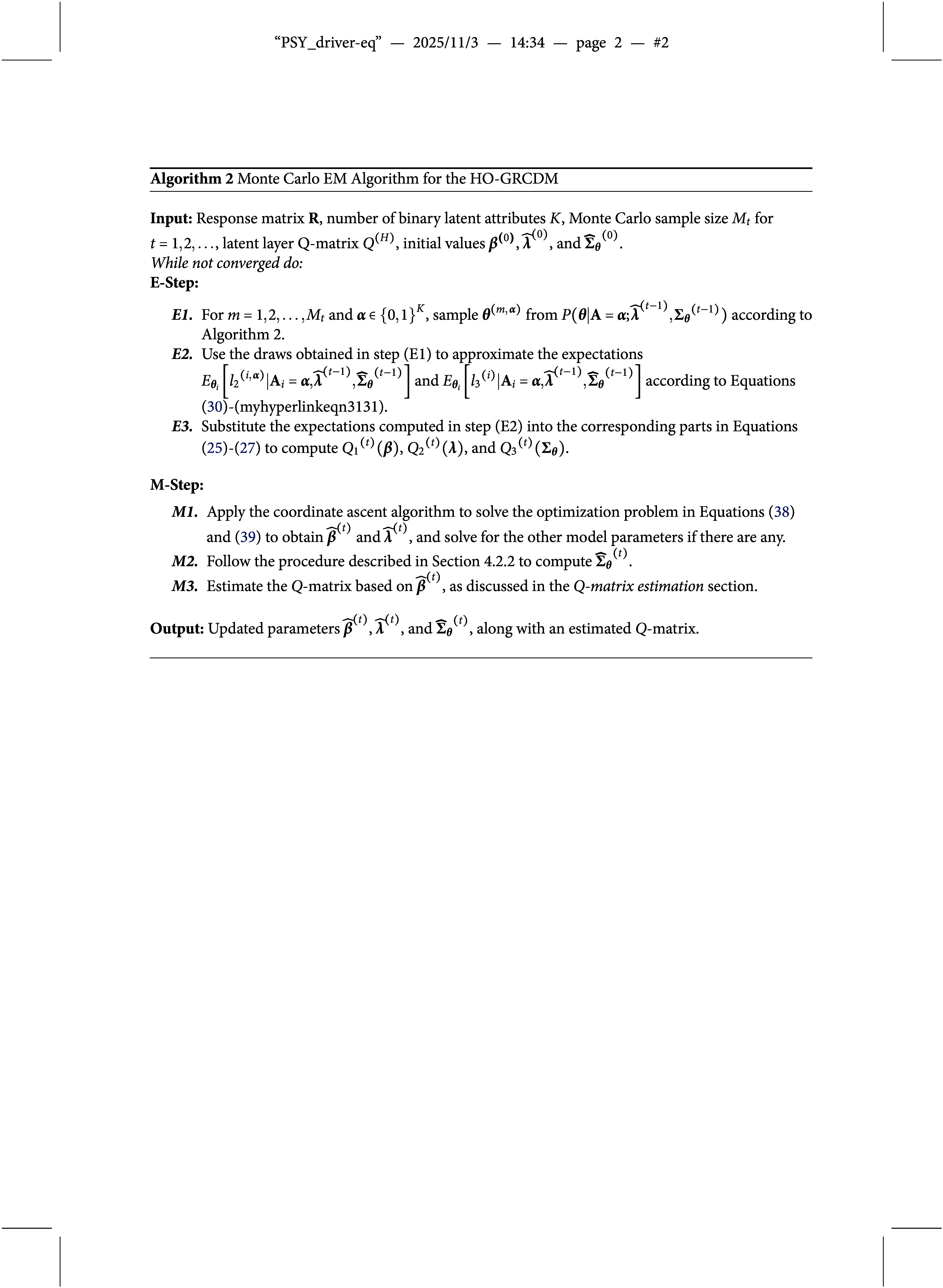

Remark 1. Many HO-GRCDMs may have additional dispersion parameters. In these cases, additional steps are needed in the M step to update these parameters explicitly or with the help of existing optimizers. For example, in the log-normal CDM case, there are additional standard deviation parameters 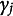 that need to be estimated. After obtaining estimates for all 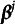 , the 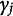 can be solved explicitly by 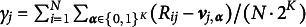 in each M step.

Remark 2. An important practical consideration when implementing the MCEM algorithm is the selection of the Monte Carlo sample size. To ensure valid statistical analysis, the numerical error from Monte Carlo approximation should be negligible relative to the statistical error, which typically decreases at a rate of 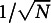 . Since numerical error depends on the Monte Carlo sample size, a larger Monte Carlo sample size is required for larger sample sizes to maintain numerical accuracy. However, increasing the Monte Carlo sample size also raises computational cost. A practical approach to balancing accuracy and efficiency is to gradually increase the Monte Carlo sample size as the algorithm progresses. This ensures that numerical precision improves as estimates stabilize while keeping early iterations computationally efficient.

Remark 3. An alternative to the MCEM algorithm proposed in this article is the Stochastic Approximation EM (SAEM; Delyon et al., 1999), which modifies the expectation approximation step. Instead of using Monte Carlo integration to approximate the expectation 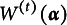 in Equation (32) at iteration *t*, SAEM employs a stochastic approximation procedure. Specifically, letting 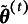 be a single sample drawn from 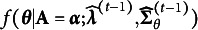 , the expectation is updated iteratively as 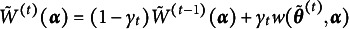 , where 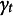 is a pre-determined step size that decreases with *t*. SAEM is computationally efficient, requiring fewer Monte Carlo samples per iteration by leveraging previously generated samples, though it relies on careful tuning of step-size parameters. In contrast, MCEM avoids the step-size tuning challenge but may demand more computational resources due to repeated sampling at each iteration. Practitioners can flexibly select between SAEM and MCEM based on specific application requirements and computational resources. Additionally, other SA-based methods for latent variable models may also be considered, such as the stochastic optimization approach discussed in Zhang & Chen (2022).

Remark 4. Initialization is an important issue for the EM algorithm. An efficient approach is to use the SVD-based algorithm to obtain the initial values (Chen et al., 2019; Zhang et al., 2020). This method leverages the low-rank nature of the design matrix to capture the principal components of the data, thus providing stable and informative starting points for the iterative fitting process. The detailed procedures for initialization are presented in the Supplementary Material.

## 5 Extension to the fully exploratory setting

In this section, we extend our identifiability theory and estimation method to the setting without a predefined higher-order latent structure. That is, no prior information about 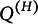 is assumed. In terms of identifiability, we present a set of identifiability conditions that are analogous to that in Section 3. In addition to the subscale/bifactor higher-order structure with an unknown 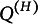 -matrix, we additionally consider an un-restricted 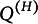 matrix (but with independent continuous factors 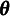 ) as well. As the individual statements resemble that in Section 3, the following paragraphs mainly describe the subscale case, and postpone additional results to Section B of the Supplemenatry Material.

The following Proposition extends Proposition 2 by considering a subscale model with an unknown 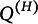 -matrix. In other words, we consider the case where the true 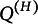 matrix is a subscale model, but do not assume any further knowledge of it. Note that *D*, the number of latent factors are still assumed to be known. As in Proposition 2, let 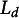 denote the attributes indexed by *k* that belong in group *d*. Additionally, assume that there are no all-zero rows in 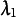 , or equivalently, 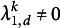 for all 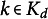 . This assumption is necessary for the exploratory setting, as it is impossible to identify the group membership when 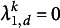 for all *d*.

Proposition 4. Consider an exploratory probit model with a subscale higher-order structure and no all-zero rows in 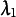 . Then, under condition C1, the model is identifiable up to label switching of the latent factors 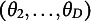 .

Now, we establish identifiability of the fully exploratory HO-GRCDM, where both *Q*-matrix and 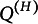 -matrix are unknown. Here, the dimensions *K* and *D* of both latent layers are assumed to be known, which is a common assumption in other identifiability results for exploratory settings (Chen et al., 2015, 2020; Lee & Gu, 2025; Xu & Shang, 2018). While being analogous to Theorem 1, the following theorem does not require the knowledge of anchor items.

Theorem 2. Consider an exploratory HO-GRCDM with a subscale higher-order structure with no all-zero rows in 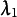 . This model is identifiable up to label switching in each layer, when the true parameters satisfy conditions A, B, and C1.

Regarding parameter estimation, the MCEM algorithm developed in Section 4 can be directly adapted to the fully exploratory HO-GRCDM scenario. The primary modification involves incorporating penalty terms into Equation (39), yielding the following modified optimization problem: 
(43)

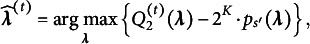



(44)

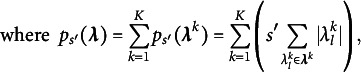

and 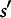 is a regularization parameter. The factor 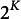 reflects the number of possible latent attribute profiles, ensuring that the penalty term is appropriately scaled relative to the likelihood. This optimization formulation closely parallels Equation (38) and can thus be solved using the same coordinate ascent approach described there.

In the case where 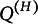 is known, the number of latent attributes *K* and that of higher-order latent factors *D* can be directly determined by 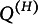 ’s dimensions. When 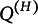 is unknown, these latent dimensions must also be inferred from data. A widely used method is to comparing models of varying dimensions with fit indices. This involves proposing a series of plausible combinations of 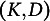 based on domain knowledge, fitting the model under each configuration, and selecting the optimal model using model-fit criteria such as BIC (Xu & Shang, 2018) and DIC (Culpepper & Chen, 2019). Despite being an important problem both from theory and method perspectives, we note that determining the optimal numbers of attributes and higher-order factors is not our primary focus. Rather, our primary contribution lies in addressing the challenging task of estimating factor loadings without structural information on the loading matrices (i.e., *Q*-matrices), given the inherent complexity and hierarchical structure of the HO-GRCDM.

## 6 Simulation studies

We conduct extensive simulation studies to investigate the performance of the proposed MCEM algorithm for HO-GRCDMs. We primarily focus on the settings with a known 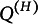 but also include a simulation study for the scenario with an unknown 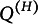 in the Supplementary Material.

We consider three sample sizes: 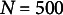 , 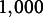 , and 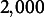 with 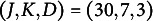 . We examine three types of models for the bottom data layer: the main-effect model, the all-effect model, and the DINA model. Within each bottom-layer model category, three types of response models are considered: (a) Bernoulli CDM for binary data, (b) Poisson CDM for count data, and (c) Lognormal CDM for continuous data. In the Bernoulli CDM case, the logistic distribution is used to model the probability of a correct response. Since both the Lognormal and Gamma distributions can be used to model positive continuous responses, we consider the Lognormal distribution as a representative case and postpone the simulation under the Gamma distribution to the Supplementary Material. The distributions for each model are detailed in Equations (4), (5), and (7). The first *K* items serve as anchor items. The bottom layer Q-matrix, 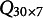 , is shown in Equation (45). For each bottom layer model, we explore two higher-order layer structures: the subscale and the bifactor structure. The true slope coefficients for the higher-order layer are also presented in Equation (45). The unconstrained parameters in 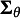 are sampled from a uniform distribution 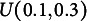 .

It is straightforward to verify that the above models are identifiable. Specifically, 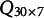 satisfies the conditions in Proposition 1, and 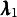 and 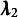 satisfy the the conditions in Propositions 2 and 3, respectively. Therefore, the identifiability conditions in Theorem 1 are satisfied. 
(45)

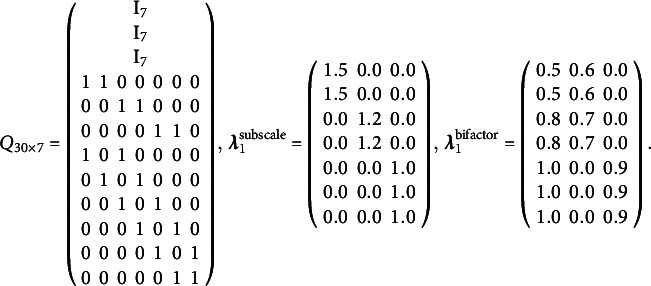

The Monte Carlo sample size 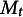 for sampling 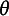 is determined by the formula 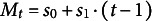 . In this study, we set 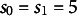 , meaning five samples are simulated in the first iteration, with the number of samples increasing by five in each subsequent iteration. The convergence criterion is 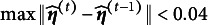 for three successive iterations. The threshold 0.04 is chosen as it yields satisfactory precision in our simulations, though a smaller value can be used for stricter convergence at the cost of increased computation time. Initialization is implemented using the SVD-based algorithm mentioned in Remark 4 and detailed in the Supplementary Material. The coordinate ascent part in our algorithm is conducted by using the R package *glmnet* (Hastie et al., 2021). For each model fitting, we apply our MCEM algorithm multiple times for a sequence of regularization parameters *s*, which vary across distributions and are listed in the Supplementary Material. The *s* that produces the smallest BIC value is then selected to finalize the model fitting.

### 6.1 Simulations for main-effect HO-GRCDMs

For the main-effect models, we set the coefficients 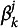 in the same way as Lee & Gu (2024): 

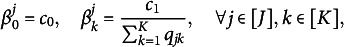

where 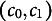 are two constants, set to ( 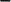 1,3) for the Lognormal-CDM, (0.5,1) for the Poisson-CDM, and ( 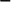 2,4) for the Bernoulli-CDM. For the Lognormal-CDM, the dispersion parameter 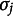 is set to 1 for all *J* items. 100 independent replications are conducted in each setting.

We report the estimation accuracy for the continuous parameters in Table 1, by displaying the Root Mean Squared Errors (RMSE) and absolute biases (aBias). Note that the differences in the bottom layer parametric families may lead to results that are not directly comparable across these models. In particular, the Bernoulli model adopts a nonlinear parametrization for the correct response probability, so its probability parameters are on a different scale than those under other models. As shown in Table 1, the estimation accuracy for all the parameters improves as the sample size increases. Furthermore, to examine the recovery of the discrete *Q*-matrix, we report the proportion of correctly estimated rows and entries in *Q* in Table 2. It can be seen that the estimation accuracy of *Q* is reasonably high and improves as the sample size grows. The simulation results in Tables 1 and 2 provide empirical verification of our identifiability results.

**Table 1:** RMSE and aBias for the main-effect HO-GRCDM

			RMSE				aBias			
Model	Higher-order structure	*N*	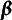	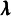	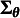	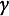	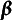	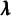	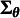	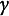
		500	0.170	0.107	0.064	0.030	0.153	0.085	0.053	0.024
		1,000	0.131	0.076	0.048	0.022	0.120	0.058	0.039	0.018
	Subscale	2,000	0.111	0.058	0.040	0.015	0.105	0.052	0.034	0.013
		500	0.166	0.240	0.178	0.032	0.148	0.188	0.164	0.026
		1,000	0.129	0.097	0.172	0.023	0.118	0.156	0.159	0.018
Lognormal	Bifactor	2,000	0.109	0.063	0.158	0.011	0.102	0.130	0.143	0.013
		500	0.265	0.345	0.164	–	0.220	0.274	0.135	–
		1,000	0.221	0.232	0.115	–	0.189	0.188	0.092	–
	Subscale	2,000	0.199	0.191	0.096	–	0.173	0.154	0.074	–
		500	0.238	0.451	0.162	–	0.201	0.348	0.146	–
		1,000	0.209	0.328	0.130	–	0.182	0.263	0.119	–
Poisson	Bifactor	2,000	0.190	0.278	0.122	–	0.172	0.227	0.113	–
		500	0.399	0.176	0.072	–	0.351	0.135	0.056	–
		1,000	0.348	0.086	0.051	–	0.316	0.067	0.042	–
	Subscale	2,000	0.313	0.083	0.041	–	0.294	0.058	0.032	–
		500	0.389	0.279	0.147	–	0.340	0.200	0.124	–
		1,000	0.344	0.189	0.132	–	0.312	0.149	0.110	–
Bernoulli	Bifactor	2,000	0.305	0.167	0.106	–	0.285	0.131	0.102	–

**Table 2:** Proportion of correctly recovered rows ( 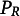 ) and entries ( 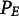 ) in *Q*-matrix for the main-effect HO-GRCDM

		Lognormal			Poisson			Bernoulli		
Higher-order structure	*N*	500	1,000	2,000	500	1,000	2,000	500	1,000	2,000
	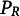	0.807	0.897	0.967	0.805	0.914	0.957	0.644	0.854	0.951
Subscale	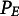	0.970	0.985	0.994	0.958	0.982	0.956	0.937	0.977	0.993
	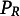	0.767	0.854	0.945	0.811	0.918	0.969	0.647	0.840	0.933
Bifactor	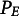	0.960	0.977	0.992	0.961	0.983	0.992	0.937	0.974	0.990

### 6.2 Simulations for all-effect HO-GRCDMs

For the all-effect models, we set the true coefficients 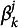 as 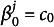 , and 

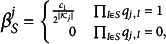

where 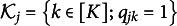 , 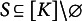 . Here, 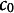 and 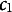 are the same constants that we have defined in Section 6.1. Similar to the main-effect model scenario, we apply the proposed MCEM algorithm to fit each model and conduct 100 independent replications for each setting. RMSE and aBias are computed for evaluating estimation accuracy.

The simulation results in Table 3 show that the estimation accuracy for all parameters improves as the sample size increases. Compared to the main-effect case, the accuracy of the continuous parameters is lower, which is unsurprising given the significantly larger number of parameters in this case. To examine the recovery of 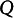 , we report the proportion of correctly estimated rows and entries in 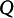 in Table 4. As explained in the *Q estimation* section, following the strict rule tends to yield an onverly dense matrix. Instead, we identify the 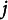 th row of 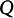 based on the largest non-zero interaction coefficient for item 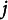 . The estimation accuracy of 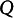 improves as the sample size grows. In fact, the recovery of 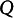 is better than in the main-effect case. This is likely due to the larger number of parameters in the all-effect case, which may help to recover the *Q*-matrix by providing more information about the dependence structure between the items and the attributes.

**Table 3:** RMSE and aBias for the all-effect HO-GRCDM

			RMSE				aBias			
Model	Higher-order structure	*N*	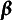	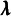	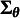	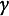	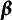	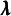	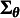	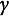
		500	0.211	0.159	0.059	0.031	0.192	0.127	0.047	0.025
		1,000	0.152	0.109	0.046	0.023	0.137	0.084	0.038	0.019
	Subscale	2,000	0.116	0.083	0.042	0.016	0.106	0.066	0.032	0.013
		500	0.191	0.209	0.143	0.031	0.170	0.167	0.131	0.025
		1,000	0.140	0.150	0.141	0.021	0.125	0.120	0.129	0.017
Lognormal	Bifactor	2,000	0.114	0.123	0.126	0.017	0.104	0.095	0.109	0.013
		500	0.310	0.258	0.072	–	0.292	0.199	0.059	–
		1,000	0.280	0.174	0.054	–	0.272	0.139	0.044	–
	Subscale	2,000	0.270	0.134	0.045	–	0.264	0.113	0.037	–
		500	0.310	0.233	0.126	–	0.293	0.187	0.110	–
		1,000	0.282	0.196	0.122	–	0.274	0.159	0.109	–
Poisson	Bifactor	2,000	0.270	0.160	0.098	–	0.266	0.133	0.080	–
		500	0.481	0.326	0.064	–	0.416	0.185	0.053	–
		1,000	0.388	0.154	0.051	–	0.346	0.105	0.038	–
	Subscale	2,000	0.335	0.062	0.040	–	0.304	0.051	0.033	–
		500	0.479	0.355	0.171	–	0.414	0.279	0.149	–
		1,000	0.393	0.191	0.106	–	0.349	0.152	0.103	–
Bernoulli	Bifactor	2,000	0.323	0.178	0.102	–	0.296	0.140	0.097	–

**Table 4:** Proportion of correctly recovered rows ( 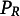 ) and entries ( 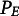 ) in *Q*-matrix for the all-effect HO-GRCDM

		Lognormal			Poisson			Bernoulli		
Higher-order structure	*N*	500	1,000	2,000	500	1,000	2,000	500	1,000	2,000
	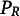	0.942	0.958	0.982	0.883	0.923	0.972	0.827	0.953	0.992
Subscale	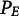	0.991	0.994	0.997	0.982	0.988	0.996	0.972	0.993	0.999
	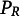	0.928	0.945	0.983	0.850	0.952	0.975	0.837	0.945	0.975
Bifactor	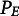	0.989	0.992	0.998	0.983	0.994	0.996	0.974	0.992	0.996

### 6.3 Simulations for DINA HO-GRCDMs

For the DINA models, we set the true coefficients 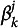 as 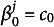 , and 

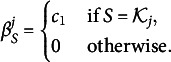

As mentioned in Section 2.1, DINA model can be regarded as a special case of the all-effect model, meaning that we can use the same estimation procedure with all-effect model to estimate DINA model. Here, we set 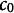 and 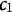 same as Section 6.1, and implement the MCEM algorithm.

These simulation results are presented in Table 5. As demonstrated in Table 5, the estimation accuracy for all parameters improves as the sample size increases. Furthermore, to examine the recovery of 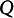 , we report the proportion of correctly estimated rows and entries in 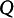 in Table 6. Since there should be only one non-zero coefficient per item, we identify the 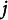 th row of 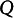 based on the largest non-zero interaction coefficient for item 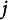 . Again, the estimation accuracy of 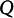 improves as the sample size grows. The results are similar to those in the all-effect case. In the confirmatory case, the DINA model has fewer parameters than the all-effect model, making it easier to estimate. However, in the exploratory case, it is fitted as an all-effect model, leading to similar computational cost. This may explain the comparable estimation accuracy in the two cases.

**Table 5:** RMSE and aBias for the DINA HO-GRCDM

			RMSE				aBias			
Model	Higher-order structure	*N*	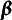	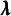	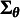	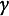	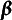	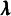	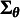	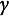
		500	0.205	0.185	0.064	0.031	0.189	0.121	0.050	0.025
		1,000	0.146	0.145	0.048	0.023	0.135	0.106	0.037	0.019
	Subscale	2,000	0.113	0.087	0.037	0.016	0.106	0.080	0.027	0.013
		500	0.204	0.218	0.171	0.033	0.189	0.168	0.159	0.027
		1,000	0.150	0.158	0.151	0.022	0.138	0.126	0.141	0.018
Lognormal	Bifactor	2,000	0.117	0.143	0.150	0.016	0.111	0.118	0.135	0.013
		500	0.387	0.196	0.081	–	0.377	0.163	0.067	–
		1,000	0.359	0.186	0.080	–	0.353	0.135	0.054	–
	Subscale	2,000	0.338	0.143	0.065	–	0.335	0.098	0.048	–
		500	0.391	0.206	0.131	–	0.380	0.166	0.120	–
		1,000	0.357	0.152	0.130	–	0.352	0.124	0.117	–
Poisson	Bifactor	2,000	0.337	0.129	0.127	–	0.334	0.102	0.114	–
		500	0.458	0.322	0.073	–	0.403	0.180	0.057	–
		1,000	0.348	0.129	0.055	–	0.316	0.101	0.042	–
	Subscale	2,000	0.320	0.109	0.036	–	0.300	0.051	0.028	–
		500	0.472	0.361	0.162	–	0.417	0.281	0.142	–
		1,000	0.383	0.208	0.158	–	0.353	0.162	0.136	–
Bernoulli	Bifactor	2,000	0.324	0.191	0.108	–	0.305	0.155	0.109	–

**Table 6:** Proportion of correctly recovered rows ( 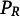 ) and entries ( 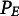 ) in *Q*-matrix for the DINA HO-GRCDM

		Lognormal			Poisson			Bernoulli		
Higher-order structure	*N*	500	1,000	2,000	500	1,000	2,000	500	1,000	2,000
	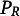	0.898	0.915	0.947	0.928	0.955	0.975	0.790	0.890	0.975
Subscale	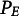	0.985	0.987	0.992	0.990	0.993	0.996	0.968	0.983	0.996
	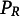	0.888	0.908	0.942	0.943	0.963	0.983	0.784	0.897	0.950
Bifactor	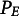	0.984	0.986	0.991	0.991	0.995	0.998	0.966	0.985	0.993

## 7 Real data analysis

In this section, we demonstrate the application of the HO-GRCDM to response time data from the 2019 TIMSS mathematics assessment (Fishbein et al., 2021). We analyze the response time data collected from United Arab Emirates students responding to booklet 1 in the eighth-grade math assessment.^1^ This dataset includes the time spent on each item screen (in seconds) by 1599 students on 28 items within a total exam time of 45 min. Data points with log response times less than 0 or greater than 6 are regarded as outliers, potentially resulting from students’ random guessing, running out of time, or taking breaks during the exam. These outliers are considered missing data (NA), and the corresponding observations are deleted, resulting in a total of 1,163 observations. The data are then transformed from seconds to minutes. A summary of descriptive information about the items is given in the Supplementary Material, including item type, item description, and the slope and location parameters obtained when fitting the two parameter item response model (2PL).

In psychometric modeling, the *Q*-matrix—or more generally, the item-attribute relationship—captures the conditional dependence of the observed outcomes associated with the items given the latent variables. While these outcome variables are typically categorical response data, they can also include process data such as response time, which reflect information into examinees’ latent traits. The use of a sparse loading matrix for response time modeling has been explored in the literature (Bolsinova & Tijmstra, 2018; He et al., 2024). In particular, He et al. (2024) adopt a same sparsity structure (analogous to the Q-matrix) for response time modeling as for response accuracy data, noting that this simplifies the interpretation of item–attribute relationships and enables simultaneous analysis of response behaviors across both dimensions. Moreover, in their empirical data analysis, they assess the benefit of including item–attribute coefficients (i.e., the sparsity structure) for response time by comparing model fit to an alternative model that removes this structure—retaining only item-level intercepts to capture general speed effects. Their DIC-based comparison suggests that incorporating item–attribute coefficients improved the overall model fit. These findings motivate us to assume the same provisional *Q*-matrices for modeling response time data as those used for response accuracy data. The TIMSS 2019 mathematics assessment is designed to measure three cognitive skills (Knowing, Applying, and Reasoning) and four content skills (Number, Algebra, Geometry, and Data and Probability), with each item specified to assess one cognitive skill and one content skill. The assessment provides provisional *Q*-matrix and 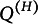 -matrix, as shown in Tables 7 and 8.

**Table 7:** *Q*-matrix of the TIMSS 2019 data set

Item	Number	Algebra	Geometry	Data and Prob.	Knowing	Applying	Reasoning
1	1	0	0	0	1	0	0
2	1	0	0	0	0	1	0
3	1	0	0	0	0	0	1
4	1	0	0	0	1	0	0
5	0	1	0	0	0	1	0
6	0	1	0	0	1	0	0
7	0	1	0	0	0	0	1
8	0	1	0	0	0	1	0
9	0	0	1	0	0	0	1
10	0	0	1	0	0	0	1
11	0	0	1	0	0	1	0
12	0	0	0	1	0	1	0
13	0	0	0	1	0	1	0
14	1	0	0	0	1	0	0
15	1	0	0	0	0	1	0
16	1	0	0	0	0	0	1
17	1	0	0	0	1	0	0
18	0	1	0	0	1	0	0
19	0	1	0	0	1	0	0
20	0	1	0	0	0	1	0
21	0	1	0	0	0	1	0
22	0	1	0	0	0	1	0
23	0	0	1	0	0	1	0
24	0	0	1	0	0	0	1
25	0	0	1	0	0	0	1
26	0	0	0	1	1	0	0
27	0	0	0	1	0	1	0
28	0	0	0	1	0	0	1

**Table 8:** 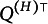
 matrix of the TIMSS 2019 data set

	Number	Algebra	Geometry	Data and Prob.	Knowing	Applying	Reasoning
Content	1	1	1	1	0	0	0
Cognitive	0	0	0	0	1	1	1

We apply the developed MCEM algorithm to fit a main-effect HO-GRCDM with a subscale higher-order structure (using the provided 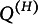 -matrix) to the dataset. We elaborate more regarding the rationale for choosing the structure of the HO-GRCDM in subsequent paragraphs. Here, note that we work under the exploratory framework with an unknown *Q*-matrix for the bottom layer, and do not use any information in the provisional *Q*-matrix. This is because our purpose is to derive findings on the test structure and item characteristics that might provide valuable insights for test developers by assessing the validity of the test design *Q*-matrix.

**Figure 1:**
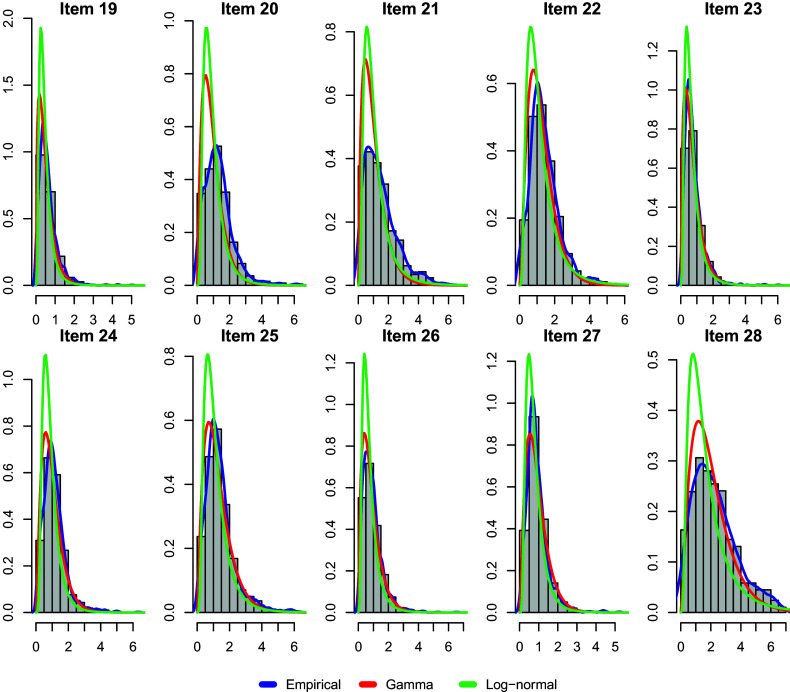
TIMSS data analysis.*Note*: Probability histogram and fitted density curves (empirical density, Gamma model, and log-normal model) for response time data (in minutes).

Regarding the choice of parametric families to model the response time, the log-normal distribution and Gamma distribution are two commonly used distributions (De Boeck & Jeon, 2019; Klein Entink et al., 2009; Maris, 1993; Van der Linden, 2006). The log-normal model is often used when the logarithm of the response times follows a normal distribution, while the Gamma model is suitable for modeling positive continuous variables with skewed distributions. To choose between these two models, we first fit both models to the data and compute their BIC values. The obtained BIC values are 73,165.46 for the Gamma model and 86,664.80 for the log-normal model. Furthermore, we plot the probability histogram for the response time of each item and fit a density curve using the spline method. Using the estimated parameters obtained by fitting the log-normal and Gamma HO-GRCDM, their corresponding density curves are also plotted. We show plots for the last 10 items (items 19–28) in Figure 1 and present plots for all items in Section F of the Supplementary Material. By examining the histogram and comparing the density curves, we found that the response time variables for most items are right-skewed, and the Gamma model’s curve (red line) overlaps more with the empirical density (blue line) than the log-normal model (green line), indicating that the Gamma model has a better fit. Therefore, we use the Gamma distribution for the response time to fit a main-effect HO-GRCDM. We conduct an additional simulation study in Section D of the Supplementary Material to investigate the performance of the MCEM algorithm for estimating the main-effect HO-GRCDM with a Gamma distribution.

We also comment on the choice of the measurement model and the latent layer structure for our HO-GRCDM. It is common to use the main-effect models to understand response times (De Boeck & Jeon, 2019; Lee & Gu, 2024; Maris, 1993; Sternberg, 1980). Lee & Gu (2024) discusses the rationale for adopting the additive model assumption in more detail. As for the high-order layer, rather than focusing on assessing general cognitive ability, our primary goal is to capture fine-grained distinctions between subskills, for which the subscale model is more appropriate.

**Figure 2:**
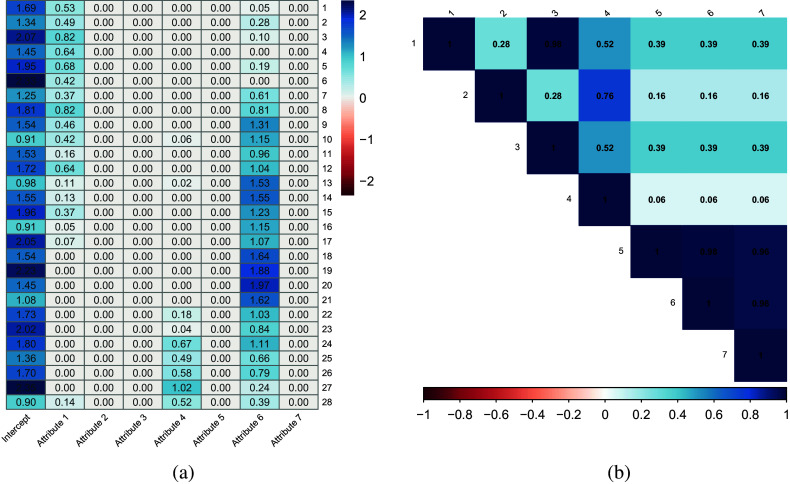
TIMSS data analysis. (a) Heatmap for estimated bottom-layer parameters. (b) Correlation plot of the estimated latent attributes.

Figure 2(a) presents heatmap of the estimated bottom-layer parameters. The fitted model reveals a sparse structure of bottom-layer coefficients—only three columns (the 1st, 4th, and 6th columns) have non-zero coefficients. In addition, there is an estimated positive correlation of 0.35 between the cognitive skill and the content skill. Using the estimated parameters, we compute 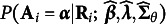 for each student, selecting the 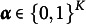 with the highest value as the estimate of their attribute profile. To explore the sparse structure and explain the three attributes with non-zero coefficients, we compute correlations among the seven attributes based on the estimated attributes of students and show the results in Figure 2(b). The first observation from the correlation plot is that the three cognitive attributes, attributes 5–7, are extremely highly correlated, with correlations up to 0.98. There are also high correlations among the four content attributes (attributes 1–4), with a correlation of 0.98 between attributes 1 and 3, and a correlation of 0.76 between attributes 2 and 4. This indicates that the content attributes may further divided into two groups: (1, 3) and (2, 4). Additionally, the correlations between one cognitive attribute and another content attribute are much smaller than that from content attributes or cognitive attributes.

The high correlations in attribute groups (1,3), (2,4), and (5,6,7) indicate that attributes within the same group are hard to distinguish. This is also reflected in the estimated bottom-layer coefficients 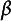 : only one attribute in each group has non-zero coefficients. The 6th column coefficients can be regarded as the coefficients for all of the cognitive skills: Knowing, Reasoning, and Applying. For attributes 1–4, we found that only items 1–15 have non-zero coefficients on the first attribute, with the majority measuring “Number” and “Algebra.” Items 24–28 have non-zero coefficients for attribute 4, and these items measure either “Geometry” or “Data and Probability.” These observations suggest that attribute groups (1,3), (2,4), and (5,6,7) correspond to (Number, Algebra), (Geometry, Data, and Probability), and (Knowing, Reasoning, Applying), respectively.

The relationships among the attributes are both intuitive and interpretable; however, the estimated coefficients do not align with the test-design-based *Q*-matrix shown in Table 7. A similar dataset was analyzed in Lee & Gu (2024) using the designed *Q*-matrix, where their findings revealed interesting patterns of intrinsic dependence among attributes. These insights motivated our application of the HO-GRCDM to the TIMSS data. To further investigate this issue, we have conducted additional analyses to explore whether a finer-grained or a coarser-grained attribute specification could improve model fit. Specifically, we fitted HO-GRCDMs in a fully exploratory scenario with five and eight attributes (i.e., 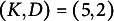 and 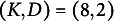 ) and compared their fit to our current HO-GRCDM model. The details of these analyses are provided below.

On the one hand, the high correlations among attributes may suggest that a model with fewer attributes would be more appropriate. Our consideration of the 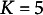 case is based on the observed significant correlations among the three cognitive attributes, indicating that they may effectively represent a single general cognitive attribute. When combined with the four content attributes, this yields a candidate model with 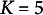 . On the other hand, incorporating more fine-grained attributes with potentially greater homogeneity within examinee groups sharing the same mastery level might better leverage the advantages of CDMs. In addition to the *Content* and *Cognitive* domain classifications, TIMSS provides *Topic Area* information, specifying the skills measured by each item. These topic areas include “Fractions and Decimals,” “Integers,” “Ratio, Proportion, and Percent,” “Expressions, Operations, and Equations,” “Relationships and Functions,” “Geometric Shapes and Measurements,” “Probability,” and “Data.” These attributes define a finer-grained test design with 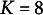 , potentially enhancing the distinction between mastery and non-mastery groups.

Regarding identifiability, neither test design necessarily guarantees an identifiable HO-GRCDM, in both the partially and fully exploratory cases. In the 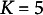 case, the higher-order model may lack identifiability due to the presence of only a single cognitive attribute. In the 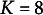 case, the topic area-derived test design does not ensure an identifiable bottom-layer model. Specifically, some attributes (e.g., “Ratio, Proportion, and Percent” and “Probability”) are measured by only a single item, making the fitted model unreliable. Despite these limitations, we consider these cases for model comparison to assess whether reducing the number of attributes ( 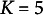 ) or adopting a finer-grained specification ( 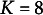 ) improves model fit. We adopt the fully exploratory case for this analysis, as it may uncover a well-fitting and interpretable model even when the specified designs are non-identifiable under the partially exploratory setting. The BIC values obtained were 73,470.34 for 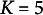 and 74,684.59 for 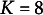 , both higher than 73,165.46 for HO-GRCDM with 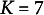 , indicating that the current 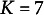 structure provides a better model fit.

Beyond this empirical comparison, we briefly discuss the broader implications of attribute specification in test development, as it directly influences the quality of data and the diagnostic insights that can be drawn from model-based analyses. One important consideration is the granularity of the attributes. As a reviewer noted, overly coarse-grained attributes may lead to considerable heterogeneity among examinees within each mastery level, which can undermine the usefulness of binary classifications and weaken the conditional independence assumption of CDMs. To address this concern, two directions may be considered. First, when possible, attributes should be defined in a fine-grained manner that realistically supports the binary mastery/non-mastery distinction and induces conditional independence among item responses. Second, in cases where attributes are inherently broad and difficult to dichotomize meaningfully, modeling them as polytomous attributes may offer a more accurate and informative representation. These considerations highlight the important role of attribute specification in ensuring the validity and interpretability of CDMs and have direct implications for future test design and development.

## 8 Discussion

We have proposed a general modeling framework, HO-GRCDM, for modeling CDMs with general responses and a higher-order structure. This framework features high flexibility in (1) addressing various types of response data, (2) adapting to a variety of measurement models, and (3) considering an exploratory settings with an unknown *Q*-matrix. Furthermore, our models have a rich representational power in its hierarchical structure to hunt for higher-order cognitive information. We provide interpretable identifiability conditions in terms of the *Q* and 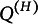 matrices that ensure the validity and accuracy of model fitting. The probit link used for the higher-order layer facilitates our identifiability theory as well as the development of an efficient MCEM algorithm for parameter estimation. Compared to existing MCMC and EM methods, our MCEM algorithm has lower computational complexity due to an explicit conditional expectation formula for 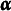 and an efficient sampler for 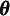 . Extensive simulation studies under various response types and measurement models are conducted to examine the efficiency of the proposed algorithm.

There are several promising directions for future work that build upon the HO-GRCDM framework. *First*, incorporating more than two layers would help explore deeper and more nuanced diagnostic information. Gu (2024) proposed a new family of DeepCDMs featuring multiple, potentially deep, entirely discrete latent layers for cognitive diagnosis. However, that work focuses on binary response variables and binary latent variables. It would be interesting to develop a framework applicable to general responses and incorporate multiple discrete latent layers. *Second*, while it is typical to consider binary attributes in CDMs, extending them to polytomous attributes can provide a more nuanced representation of latent skills (Chen & de la Torre, 2013; Karelitz, 2004; von Davier, 2005). Third, the model can be further extended by jointly modeling multiple types of responses (Man & Harring, 2023; Van der Linden, 2007; Wang, Zhang, et al., 2018). Many assessments include a mix of binary, count, and continuous responses, each providing complementary diagnostic information. Extending HO-GRCDM to accommodate such heterogeneous responses while preserving its higher-order structure would enhance its flexibility and practical utility.

## Supporting information

Liu et al. supplementary material Liu et al. supplementary material

## Footnotes

1 The data and related information are available from https://timssandpirls.bc.edu/timss2019/.

## Supplementary material

The supplementary material for this article can be found at https://doi.org/10.1017/psy.2025.15.
