# Supplementary material for: Exploratory General-Response Cognitive Diagnostic Models with Higher-Order Structures
Source: Psychometrika. 2025 Apr 16;90(5):1651–78. doi: 10.1017/psy.2025.15 (PMC12805203; doi:10.1017/psy.2025.15)
Supplement: Liu et al. supplementary material [file S0033312325000158sup001.pdf]

Supplementary Material to "Exploratory General-response Cognitive Diagnostic Models with Higher-order Structures"

Jia Liu*† Seunghyun Lee* Yuqi Gu*

*Department of Statistics, Columbia University
†School of Mathematics and Statistics, Northeast Normal University

Section A provides the proofs of the identifiability results under the partially exploratory setting discussed in Section 3 of the main text. Section B presents additional theoretical results and proofs for identifiability under the fully exploratory model setting. Section C outlines the initialization strategy based on the singular value decomposition. Section D offers additional details for the simulation studies described in Section 6 of the main text. Section E reports a simulation study for the fully exploratory HO-GRCDM introduced in Section 5 of the main text. Finally, Section F provides supplementary details for the real data analysis.

A Proof of Partially Exploratory Identifiability Results

Proof of Proposition 1. The conclusion of this proposition directly follows from that of Proposition 1 in Lee and Gu (2025), by recognizing that the deep discrete encoder model proposed in that paper reduces to the general-response CDM when marginalizing out all of the deeper discrete latent layers except the first latent layer. □

Proof of Proposition 2. We separate the arguments for sufficiency and necessity. Recall that for each d, we assume that there exists a *pivot row* k_d such that $\lambda_{1,d}^{k_d} > 0$.

This research was conducted when Jia Liu was a visiting PhD student in the Department of Statistics at Columbia University.

Sufficiency. We prove that the model is identifiable under the given condition. For each $k \in [K]$, let Z_k denote the unique group that the kth item belongs to, and define $\delta_k := \frac{\lambda^k_{1,Z_k}}{\sqrt{1+(\lambda^k_{1,Z_k})^2}}$. It is clear that $\lambda \mapsto \frac{\lambda}{\sqrt{1+\lambda^2}}$ is a one-to-one mapping. Hence, using Proposition 2 in Fang et al. (2021), it suffices to show that one can uniquely recover $\delta = (\delta_1, \ldots, \delta_K)$ and Σ, given the values

$$C_\rho(k,l) = \frac{\lambda_1^{k\top}\Sigma\lambda_1^l}{\sqrt{1+\lambda_1^{k\top}\Sigma\lambda_1^k}\sqrt{1+\lambda_1^{l\top}\Sigma\lambda_1^l}} = \sigma_{Z_kZ_l}\delta_k\delta_l, \text{ for } k \neq l.$$

Fix any d. We first prove that δ_k for $k \in L_d := \{k \in [K] : Z_k = d\}$ can be identified. Let us first consider the first scenario with $K_d \geq 3$. For $k \neq l \in L_d$, we have $Z_k = Z_l = d$. Because $\sigma_{dd} = 1$, $C_\rho(k,l)$ simplifies into $\delta_k\delta_l$. Using the fact that $\delta_{k_d} > 0$ for the pivot row indexed by k_d, we can uniquely determine all δ_k for $k \in L_d$. To see this, one can simply take two additional indices $l \neq m \in L_d$ and consider the equations

$$C_\rho(k_d,l) = \delta_{k_d}\delta_l, \quad C_\rho(k_d,m) = \delta_{k_d}\delta_m, \quad C_\rho(l,m) = \delta_l\delta_m.$$

Next, we consider the case when $K_d = 2$ and $\sigma_{dd'} \neq 0$ for some $d' \neq d$. Since $\delta_{k_{d'}} \neq 0$ and $\sigma_{dd'} \neq 0$, one can recover the ratio of δ_ks for $k \in L_d = \{k_d, k_d+1\}$ by computing the ratio of $C_\rho(k,k_{d'}) = \sigma_{dd'}\delta_k\delta_{k_{d'}}$'s. Then, we can uniquely determine δ_k's for $k \in L_d$ using the value of $C_\rho(k_d,k_d+1) = \delta_{k_d}\delta_{k_d+1}$.

Finally, it remains to recover the off-diagonal entries of Σ. But this directly follows by noting that $\sigma_{dd'} = \frac{C_\rho(k_d,k_{d'})}{\delta_{k_d}\delta_{k_{d'}}}$ for any $d \neq d'$. Here, note that $\delta_{k_d}\delta_{k_{d'}} \neq 0$ by the pivot row assumption, so the fraction is always well-defined.

Necessity. We show that the conditions in the theorem are necessary for identifiability using the proof by contradiction. Suppose an identifiable model has a d such that (1) $K_d = 1$ or (2) $K_d = 2$ and $\sigma_{dd'} = 0$ for all $d' \neq d$. Under the first case with $K_d = 1$, the d-th group is only measured by the k_d-th item. Then, the parameters $\delta_{k_d}, \{\sigma_{dd'}\}_{d' \neq d}$ are only reflected in $C_\rho(k_d,l) = \sigma_{dZ_l}\delta_{k_d}\delta_l$'s, for $l \neq k_d$. Even when assuming that the values of δ_l's are known, this is a system of $D-1$ equations with D unknowns, and does not exhibit a unique solution. Hence, we have a contradiction.

Next, we consider the case where $K_d = 2$ and $\sigma_{dd'} = 0$ for all $d' \neq d$. Then, $L_d = \{k_d, k_d + 1\}$. Because $\sigma_{dd'} = 0$ for $d' \neq d$, $\rho_{kl} = 0$ for all $k \in L_d$. Hence, δ_{k_d} and δ_{k_d+1} needs to be determined only by the value of $C_\rho(k_d, k_d + 1) = \delta_{k_d}\delta_{k_d+1}$. This is a single equation with two unknowns and not solvable, giving the contradiction. □

Proof of Theorem 1. The proof follows by sequentially applying the identifiability results for the CDM and the higher-order probit model in a layerwise manner. First, we consider the bottom layer CDM with parameters $(\boldsymbol{\pi}, \boldsymbol{\beta}, Q)$. Here, we are setting $\pi_{\boldsymbol{\alpha}} = P(\mathbf{A} = \boldsymbol{\alpha})$ by marginalizing out the probit latent layer using eq. (14), as mentioned in the main text. Then, under conditions A and B, Proposition 1 gives the identifiability of the CDM parameters $(\boldsymbol{\pi}, \boldsymbol{\beta}, Q)$, up to latent variable permutation. As we assume the knowlege of anchor items for each latent variable, we can identify these parameters without any trivial ambiguity.

Now, having identified $\boldsymbol{\pi}$, we fully know the marginal distribution of the latent variables. Now, we can apply Propositions 2 or 3 to identify the probit model parameters $(\Sigma_\theta, \boldsymbol{\lambda}_0, \boldsymbol{\lambda}_1)$ and the proof is complete. Note that the conditions for the probit parameters in Theorem 1 are exactly those in Propositions 2 or 3. □

B Identifiability of Fully Exploratory Models: Additional Results and Their Proofs

B.1 Additional identifiability results

Continuing from Section 5, this supplement provides additional identifiability results when the upper-layer $Q^{(H)}$-matrix is unknown. In additional to the subscale higher-order model that was presented in the main text, we consider two additional sets of assumptions: (1) $\Sigma_\theta = I_K$ but no restrictions on $Q^{(H)}$, and (2) bifactor model. All related proofs (including that of Proposition 4 in the main text) are presented in the next subsection.

First, we work under the most general setup without any assumptions on $Q^{(H)}$. Then, there exists a trivial rotation ambiguity regarding the covariance matrix Σ_θ, as the item parameters are

only determined up to $\lambda_1 \Sigma_\theta^{1/2}$. Thus, we further assume that $\Sigma_\theta = I_K$. This problem has been recently addressed in the context of probit models in Li et al. (2025), and we summarize the result below. Here, condition D1 is an analog of the popular condition in factor analysis that guarantees identifiability up to rotation, originally introduced in the seminal work of Anderson and Rubin (Anderson and Rubin, 1956). Condition D2 is a convenient assumption for fixing the rotation and also the label switching, and we expect that it may be possible to replace it with a weaker criteria.[1]

Proposition 5 (Theorem 3.3 in Li et al. (2025))**.** *The exploratory probit model with parameters λ_0, λ_1 is identifiable up to label switching when the following condition holds. Here, $\bar{\lambda}_1 := [\bar{\lambda}_1^1, \ldots, \bar{\lambda}_1^K]^\top$ is a $J \times K$ matrix, where $\bar{\lambda}_1^k := \frac{\lambda_1^k}{\sqrt{1+\|\lambda_1^k\|^2}}$.*

D1. If any row of $\bar{\lambda}_1$ is deleted, there exist two disjoint submatrices with rank K.

D2. The matrix λ_1 is lower triangular, after a column permutation.

Next, we provide a similar result under the bifactor model. Similar to the main text, we assume that $\Sigma_\theta = \begin{pmatrix} 1 & \mathbf{0}^\top \\ \mathbf{0} & \Sigma_\theta^\star \end{pmatrix}$ to avoid trivial ambiguities. Here, $\Sigma_\theta^\star$ is a $(D-1) \times (D-1)$ matrix whose diagonal entries are all ones. Also, recall that we have defined L_d for each $d \in [D-1]$ as the attributes that belong in group d. Similar to the subscale case, we assume that all group-specific effects are nonzero (i.e. $\lambda_{1,d+1}^k \neq 0$ for $k \in L_d$) to avoid trivial indeterminacy.

Proposition 6. *Consider an exploratory probit model with a bifactor higher-order structure. Then, the model is identifiable up to label switching when (i) eq. (16) holds (or equivalently, $|L_d| \geq 3$), (ii) there exists at least two groups such that the matrix $\lambda_{1,:}^{L_d}$ has at least three rows that are not multiples of each other, and (iii) at least one of condition C3 or C4 holds.*

We postpone the proof of the above proposition to Section B.2.

Now, by combining above two propositions, we have the following conclusion guaranteeing the identifiability of fully exploratory HO-GRCDMs. This result is similar to Theorem 2 in the main paper, which focused on the subscale case. Here, part (b) generalizes Theorem 1 in the main

[1]Assumption D2 is satisfied for the subscale model, but not for the bifactor model.

text, at the cost of assuming stronger conditions. We omit the proofs as it is identical to that of Theorem 1.

Theorem 3. *Consider a fully exploratory HO-GRCDM. The following three conclusions hold.*

(a) Suppose $\Sigma_\theta = I_D$ is known. Then, the HO-GRCDM is identifiable up to label switching in each layer, when the true parameters satisfy conditions A, B, D1, and D2.

*(b) Assume a **bifactor** higher-order structure with no all-zero rows in λ_1. The HO-GRCDM is identifiable up to label switching in each layer, when the true parameters satisfy conditions A, B, and all three conditions in Proposition 6.*

B.2 Proof of Propositions 4 and 6

Proof of Proposition 4. Suppose that there exists an alternative set of parametrs $(\tilde{\lambda}_0, \tilde{\lambda}_1, \tilde{Q}^{(H)}, \tilde{\Sigma})$ that define the same tetrachoric correlations. For the sake of notational convenience, we omit the subscript θ from Σ_θ in this proof. Let us extend the definition of the $K \times D$ matrix $\bar{\lambda}_1$ in Proposition 5 to a general covariance matrix Σ, by defining the kth row as

$$\bar{\lambda}_1^k := \frac{\lambda_1^k \Sigma^{1/2}}{\sqrt{1 + \lambda_1^{k\top} \Sigma \lambda_1^k}}.$$

Here, $\Sigma^{1/2}$ is the unique positive definite square root of Σ. Then, under the assumption that $|K_d| \geq 3$, $\bar{\lambda}_1$ satisfies the row-deletion condition D1. Hence, an application of Anderson and Rubin's argument for linear factor analysis (see Theorem 3.3 of Li et al. (2025)) shows that we must have $\tilde{\lambda}_0 = \lambda_0$, and that $\lambda_1 \Sigma^{1/2}$ is determined up to a $K \times K$ rotation matrix R multiplied on the right, in other words

$$V = \lambda_1 \Sigma^{1/2} = \tilde{\lambda}_1 \tilde{\Sigma}^{1/2} R. \tag{46}$$

For notational simplicity, let $s_1, \ldots, s_D$ be the row-vectors of $\Sigma^{1/2}$. Note that $\Sigma^{1/2}$ non-singular, so $s_1, \ldots, s_D$ are linearly independent.

Under the subscale assumption, each row of λ_1 and $\tilde{\lambda}_1$ must have exactly one non-zero entry. Thus, all rows of V indexed by $k \in K_d$ must be parallel to s_d and nonzero. In other words, these row vectors form a straight line, and this line is distinct for each d. Now, looking at the alternative parameters with $VR^{-1} = \tilde{\lambda}_1\tilde{\Sigma}^{1/2}$, the straight line structure does not change under the rotation R^{-1}. Consequently, we can cluster identical groups for the alternative parameters, by defining an equivalence relation $k \sim l$ if and only if the kth and lth row of V are parallel. Here, each $k \in [K]$ is clustered into a unique group, as we assume that $\lambda_{1,d}^k \neq 0$ for some d. Hence, $Q^{(H)}$ and $\tilde{Q}^{(H)}$ are identical up to label switching (i.e. identical up to column permutation).

Without loss of generality, suppose that $Q^{(H)} = \tilde{Q}^{(H)}$. Then, we are back to the confirmatory subscale model, where we have already showed identifiability in Proposition 2. Hence, the proof is complete. □

Remark. Note that we only require $K_d \geq 3$ to satisfy condition D1. It would be interesting to study if the confirmatory necessary and sufficient conditions generalize to the exploratory case as well.

Proof of Proposition 6. Suppose that there exists an alternative set of parameters $(\tilde{\lambda}_0, \tilde{\lambda}_1, \tilde{Q}^{(H)}, \tilde{\Sigma})$ that define the same tetrachoric correlations. We also define $\tilde{L}_d$ similar to L_d, but using the alternative paramter $\tilde{\lambda}_1$. As we assume $|L_d| \geq 3$ and that all group-specific effects are nonzero, the row deletion condition D1 holds. By the same argument as the proof of the previous Theorem, we have $\lambda_0 = \tilde{\lambda}_0$ and (46).

Let v_k denote the kth row of V, where V is the matrix defined in (46). Also, let $s_1, \ldots, s_D$ denote the rows of $\Sigma^{1/2}$, and let $\tilde{s}_1, \ldots, \tilde{s}_D$ denote the rows of $\tilde{\Sigma}^{1/2}R$. It is clear that $(s_1, \ldots, s_D)$ are linearly independent, as well as $(\tilde{s}_1, \ldots, \tilde{s}_D)$. As we assume $\Sigma = \begin{pmatrix} 1 & \mathbf{0}^\top \\ \mathbf{0} & \Sigma^\star \end{pmatrix}$, we must have $s_1 = (1, \mathbf{0}_{D-1}^\top)^\top$.

We claim that for each $k \neq l$, $\operatorname{rank}(s_1, v_k, v_l) = 2$ if and only if k, l belong in the same group, under both the true and alternative parametrization. First, let us consider the true parametrization representation for $V = \lambda_1\Sigma^{1/2}$. The "if" part holds because the bifactor structure of λ_1 implies that for all $k \in L_d$, v_k must be a linear combination of s_1 and s_{d+1}. The "only if" part follows by a proof by contradiction. Suppose $\operatorname{rank}(s_1, v_k, v_l) = 2$ but k and l belong in distinct groups, say d and d'. Then, we can write $v_k = \lambda_{1,1}^k s_1 + \lambda_{1,d+1}^k s_d$ and $v_l = \lambda_{1,1}^l s_1 + \lambda_{1,d'+1}^l s_{d'}$, and we have a contradiction

because $s_1, s_d, s_{d'}$ are linearly independent.

Next, we prove the claim under the alternate parameters with $V = \tilde{\lambda}_1 \tilde{\Sigma}^{1/2} R$. It suffices to show that $\tilde{s}_1$, the first row of $\tilde{\Sigma}^{1/2} R$, is parallel to s_1. If so, $\text{rank}(s_1, v_k, v_l) = \text{rank}(\tilde{s}_1, v_k, v_l)$ and the argument in the above paragraph directly applies. To prove this claim, we use the assumption that there exists two (true) groups, say d and e, such that $\lambda_{1,d+1}^{L_d}$ (and $\lambda_{1,e+1}^{L_e}$) have at least three rows indexed by k_d, l_d, m_d (and k_e, l_e, m_e) that are not multiples of each other. For notational simplicity, let $U_d := \text{span}(v_{k_d}, v_{l_d}, v_{m_d})$ and $U_e := \text{span}(v_{k_e}, v_{l_e}, v_{m_e})$. Then, we must have $\text{rank}(U_d) = \text{rank}(U_e) = 2$. Also, as $U_d \neq U_e$ and $s_1 \in U_d, U_e$, we have $U_d \cap U_e = \mathbb{R}s_1 := \{cs_1 : c \in \mathbb{R}\}$. The rank of U_d and U_e implies that $k_d, l_d, m_d \in \tilde{L}_{d'}$ and $k_e, l_e, m_e \in \tilde{L}_{e'}$ for some $d' \neq e'$ (i.e. belong in a same alternative group), hence $U_d = \text{span}(\tilde{s}_1, \tilde{s}_{d'+1})$ and $U_e = \text{span}(\tilde{s}_1, \tilde{s}_{e'+1})$. Consequently, we have $U_d \cap U_e = \mathbb{R}\tilde{s}_1$ and s_1 is parallel to $\tilde{s}_1$.

Now, we can cluster $[K]$ into $D-1$ groups by computing $\text{rank}(s_1, v_k, v_l)$ for each pair $k \neq l \in [K]$. The resulting groups must be identical under the true and alternative parameters, up to a trivial label switching. In other words, $Q^{(H)}$ and $\tilde{Q}^{(H)}$ are identical up to column permutations. The proof is complete by applying the confirmatory result in Proposition 3. □

C Initialization Procedure for Simulation Studies

C.1 Initialization of Bottom Layers for Various Response Distributions

We use singular value decomposition (SVD) to find the starting values for the bottom layers. We first start at the Bernoulli model case and present Algorithm 3 for binary data as below.

Algorithm 3 is based on the SVD-based estimator in Zhang et al. (2020). It utilizes SVD twice. The initial application of SVD, followed by the inverse transformation (Steps 2-5), serves to denoise and linearize the data. Subsequently, the second application of SVD (Steps 6-7) performs PCA on the linearized data. For further discussions on the details, please refer to Zhang et al. (2020) and Chatterjee (2015). The difference from Zhang et al. (2020) is that we apply the Varimax rotation to achieve a sparse and more interpretable factor loading structure in Step 8.

We utilize initialization for the HO-GRCDMs with other general responses based on a similar

Algorithm 3 Initialization for Bernoulli models

1. Input response data $\mathbf{R} = (r_{ij})_{N\times J}$, number of attributes K, link function g, and truncation parameter $\varepsilon_{N,J} > 0$.

2. Apply the singular value decomposition to $\mathbf{R} = \sum_{j=1}^{J} \tau_j \mathbf{u}_j \mathbf{v}_j^\top$, where $\tau_1 \geq \tau_2 \geq \ldots \tau_J$ are the singular values, and $\mathbf{u}_j$s and $\mathbf{v}_j$s are left and right singular vectors, respectively.

3. Let $\mathbf{X} = (x_{ij})_{N\times J} = \sum_{k=1}^{\tilde{K}} \tau_k \mathbf{u}_k \mathbf{v}_k^\top$, where $\tilde{K} = \max\left\{K+1, \arg\max_{\mathrm{k}}\left\{\tau_{\mathrm{k}} \geq 1.01\sqrt{\mathrm{N}}\right\}\right\}$

4. Let $\widehat{\mathbf{X}} = (\widehat{x}_{ij})_{N\times J}$ be defined as

$$\widehat{x}_{ij} = \begin{cases} \varepsilon_{N,J} & if \quad x_{ij} < \varepsilon_{N,J} \\ x_{ij} & if \quad \varepsilon_{N,J} \leq x_{ij} \leq 1-\varepsilon_{N,J} \\ 1-\varepsilon_{N,J} & if \quad x_{ij} \geq 1-\varepsilon_{N,J} \end{cases}$$

5. Let $\tilde{\mathbf{M}} = (\tilde{m}_{ij})_{N\times J}$, where $\tilde{m}_{ij} = g(\widehat{x}_{ij})$.

6. Let $\widehat{\beta}_0 = (\widehat{\beta}_0^1, \ldots, \widehat{\beta}_0^J)$, where $\widehat{\beta}_0^j = (\sum_{i=1}^{N} \tilde{m}_{ij})/N$.

7. Apply singular value decomposition to $\widehat{\mathbf{M}} = (\tilde{m}_{ij} - \widehat{\beta}_0^j)_{N\times J}$ to have $\widehat{\mathbf{M}} = \sum_{j=1}^{J} \widehat{\tau}_j \widehat{\mathbf{u}}_j \widehat{\mathbf{v}}_j^\top$, where $\widehat{\tau}_1 \geq \widehat{\tau}_2 \geq \ldots \widehat{\tau}_J$ are the singular values, and $\widehat{\mathbf{u}}_j$s and $\widehat{\mathbf{v}}_j$s are left and right singular vectors, respectively.

8. Apply varimax to $\widehat{\mathbf{V}} = (\widehat{\mathbf{v}}_1, \ldots, \widehat{\mathbf{v}}_J)$, and let $\tilde{\mathbf{V}}$ be the rotated version of $\widehat{\mathbf{V}}$.

9. Output $\widehat{\beta} = (\beta_k^j)_{J\times K} = \frac{1}{\sqrt{N}}(\widehat{\tau}_1 \tilde{\mathbf{v}}_1, \ldots, \widehat{\tau}_K \tilde{\mathbf{v}}_K)$, $\widehat{\beta}_0$.

idea to that of Algorithm 3. Firstly, the procedure is simpler and more directly for Transformed-normal distribution. Let T_{ij} denote the transformed response variable. For example, $T_{ij} = \log(R_{ij})$ for log-normal distribution, $T_{ij} = \log(R_{ij}/(1-R_{ij}))$ for logistic-normal distribution, and so forth. After transforming R_{ij} to T_{ij}, there is no need to linearize data and truncate variable. The procedure of finding the starting points for Transformed-normal distributions is listed in Algorithm 4.

Algorithm 4 Initialization for Transformed-Normal models

1. Input transformed response data $\mathbf{T} = (t_{ij})_{N\times J}$, number of attributes K, link function g.
2. Apply the singular value decomposition to $\mathbf{T} = \sum_{j=1}^{J} \tau_j \mathbf{u}_j \mathbf{v}_j^\top$, where $\tau_1 \geq \tau_2 \geq \ldots \tau_J$ are the singular values, and $\mathbf{u}_j$s and $\mathbf{v}_j$s are left and right singular vectors, respectively.
3. Let $\mathbf{X} = (x_{ij})_{N\times J} = \sum_{k=1}^{\tilde{K}} \tau_k \mathbf{u}_k \mathbf{v}_k^\top$, where $\tilde{K} = \max\left\{K+1, \arg\max_{\mathrm{k}}\left\{\tau_{\mathrm{k}} \geq 1.01\sqrt{\mathrm{N}}\right\}\right\}$, compute $\widehat{\beta}_0^j = (\sum_{i=1}^{N} x_{ij})/N$, $j = 1,\ldots,J$.
4. Apply varimax to $\widehat{\mathbf{V}} = (\mathbf{v}_1,\ldots,\mathbf{v}_J)$, and let $\tilde{\mathbf{V}} = (\tilde{\mathbf{v}}_1,\ldots,\tilde{\mathbf{v}}_J)$ be the rotated version of $\widehat{\mathbf{V}}$.
5. Output $\widehat{\beta} = (\beta_k^j)_{J\times K} = \frac{1}{\sqrt{N}}(\tau_1\tilde{\mathbf{v}}_1,\ldots,\tau_K\tilde{\mathbf{v}}_K)$, $\widehat{\beta}_0 = (\widehat{\beta}_0^1,\ldots,\widehat{\beta}_0^J)$.

The initialization of Poisson models presented in Algorithm 5. It is similar to the Bernoulli case. The difference is SVD is applied to transformed data $T_{ij} = \log(R_{ij}+1)$ instead of the original data R_{ij} to help stabilize the variance and reduce skewness, then the data is transformed back to the original scale in step 5.

Algorithm 5 Initialization for Poisson models

1. Input response data $\mathbf{R} = (r_{ij})_{N\times J}$, number of attributes K, link function g, and truncation parameter $\varepsilon_{N,J} = \log(1)$.

2. Transforming the data $\mathbf{R}$ to $\mathbf{T} = (t_{ij})_{N\times J}$, with $t_{ij} = \log(R_{ij}+1)$. Apply the singular value decomposition to $\mathbf{T} = \sum_{j=1}^{J} \tau_j \mathbf{u}_j \mathbf{v}_j^\top$, where $\tau_1 \geq \tau_2 \geq \ldots \tau_J$ are the singular values, and $\mathbf{u}_j$s and $\mathbf{v}_j$s are left and right singular vectors, respectively.

3. Let $\mathbf{X} = (x_{ij})_{N\times J} = \sum_{k=1}^{\tilde{K}} \tau_k \mathbf{u}_k \mathbf{v}_k^\top$, where $\tilde{K} = \max\left\{K+1, \arg\max_{\mathrm{k}}\left\{\tau_{\mathrm{k}} \geq 1.01\sqrt{\mathrm{N}}\right\}\right\}$

4. Let $\widehat{\mathbf{X}} = (\widehat{x}_{ij})_{N\times J}$ be defined as

$$\widehat{x}_{ij} = \begin{cases} \varepsilon_{N,J} & if \quad x_{ij} < \varepsilon_{N,J} \\ x_{ij} & if \quad x_{ij} \geq \varepsilon_{N,J} \end{cases}$$

5. Let $\tilde{\mathbf{M}} = (\tilde{m}_{ij})_{N\times J}$, where $\tilde{m}_{ij} = \exp(\widehat{x}_{ij}) - 1$.

6. Compute $\widehat{\beta}_0^j = (\sum_{i=1}^{N} \tilde{m}_{ij})/N$, $j = 1, 2, \ldots, J$.

7. Apply singular value decomposition to $\widehat{\mathbf{M}} = (\tilde{m}_{ij} - \widehat{\beta}_0^j)_{N\times J}$ to have $\widehat{\mathbf{M}} = \sum_{j=1}^{J} \widehat{\tau}_j \widehat{\mathbf{u}}_j \widehat{\mathbf{v}}_j^\top$, where $\widehat{\tau}_1 \geq \widehat{\tau}_2 \geq \ldots \widehat{\tau}_J$ are the singular values, and $\widehat{\mathbf{u}}_j$s and $\widehat{\mathbf{v}}_j$s are left and right singular vectors, respectively.

8. Apply varimax to $\widehat{\mathbf{V}} = (\widehat{\mathbf{v}}_1, \ldots, \widehat{\mathbf{v}}_J)$, and let $\tilde{\mathbf{V}} = (\tilde{\mathbf{v}}_1, \ldots, \tilde{\mathbf{v}}_J)$ be the rotated version of $\widehat{\mathbf{V}}$.

9. Output $\widehat{\beta} = (\beta_k^j)_{J\times K} = \frac{1}{\sqrt{N}}(\widehat{\tau}_1 \tilde{\mathbf{v}}_1, \ldots, \widehat{\tau}_K \tilde{\mathbf{v}}_K)$, $\widehat{\beta}_0 = (\widehat{\beta}_0^1, \ldots, \widehat{\beta}_0^J)$.

C.2 Initialization of Higher-Order Layers

Once the initial values for the bottom layer are obtained, the conditional probability $P(\mathbf{R}_i^j|\alpha,\beta^j)$ for each model can be computed according to Equations (5)-(9). For each $\alpha \in \{0,1\}^K$, we can then compute the normalized likelihood of $\boldsymbol{\alpha}$,

$$P_\alpha = \frac{\prod_{i=1}^N P(\mathbf{R}_i^j|\alpha,\beta^j)}{\sum_{\alpha'} \prod_{i=1}^N P(\mathbf{R}_i^j|\alpha',\beta^j)}, \tag{47}$$

and use it as an initial approximation of $\pi_{\boldsymbol{\alpha}}$, the marginal proportion of the attribute pattern $\boldsymbol{\alpha}$. The approach of approximating marginals via normalized likelihood terms is well-recognized in Bayesian inference (Gelman et al., 2013) and in probabilistic modeling (Bishop and Nasrabadi, 2006).

Using P_α as an initial approximation of $\pi_{\boldsymbol{\alpha}}$, we generate a set of pseudo-attribute data according to the distribution $\{P_\alpha : \boldsymbol{\alpha} \in \{0,1\}^K\}$. This step aims to find appropriate starting points for $\lambda_{\mathbf{1}}{}^k$ and λ_0^k in the following model:

$$P(A_k = 1|\theta, \lambda_{\mathbf{1}}{}^k, \lambda_0^k) \;\; = \;\; f^{-1}(\theta^\top \lambda_{\mathbf{1}}{}^k + \lambda_0^k), \quad k = 1,\ldots,K. \tag{48}$$

Given the generated pseudo-attribute data $\mathbf{A}_{pseudo}$, this process is reduced to finding starting points for an item factor analysis model with a probit link $\Phi(\cdot)$, for which Algorithm 3 can be used to obtain the initial values. The complete initialization procedure is given in Algorithm 6. Note that, the sample size of $\mathbf{A}_{pseudo}$ is flexible and is not necessary to align with the response data size N. A larger number of $\mathbf{A}_{pseudo}$ will help ensure that the pseudo-attribute data distribution is sufficiently close to P_α, while a smaller number of $\mathbf{A}_{pseudo}$ can render larger randomness but enable faster computation if N is very large.

Algorithm 6 Complete Initialization Procedure

1. Response data $\mathbf{R} = (r_{ij})_{N\times J}$, number of attributes K, and link function g.
2. Based on the response type, apply one of Algorithms 3-5 accordingly to find initial values of $\widehat{\beta}$.
3. Compute P_α according to Equation (47).
4. Generate a set of pseudo-attribute data $\mathbf{A}_{pseudo}$ according to P_α.
5. Impute $\mathbf{A}_{pseudo}$ into Equation (48) and apply Algorithm 3 to get the initial values of λ, using the probit link function $\Phi(\cdot)$.
6. Output the initial values $\widehat{\beta}$ and $\widehat{\lambda}$.

D Simulation Study Details

D.1 Simulation Details

Table 9 presents the sequences of the tuning parameters s for different sample sizes and models in the simulation study. The sequences are chosen to decrease as the sample size increases, following the theoretical suggestion for regularization parameter selection Chen et al. (2015). The magnitude of the sequences differs across models because of variations in the true parameter values.

Sample size	Model			
	Lognormal	Poisson	Bernoulli	Gamma
500	$(0.10, 0.12, 0.14)$	$(0.08, 0.09, 0.10)$	$(0.022, 0.023, 0.024)$	$(0.08, 0.09, 0.10)$
1000	$(0.08, 0.10, 0.12)$	$(0.07, 0.08, 0.09)$	$(0.020, 0.021, 0.022)$	$(0.08, 0.09, 0.10)$
2000	$(0.06, 0.08, 0.10)$	$(0.06, 0.07, 0.08)$	$(0.018, 0.019, 0.020)$	$(0.06, 0.07, 0.08)$

Table 9: Sequences of tuning parameters s for different sample sizes and models (Lognormal, Poisson, Bernoulli, and Gamma) used in the simulation study.

Model	Higher-Order Structure	N	RMSE				aBias			
			β	λ	Σ_θ	s	β	λ	Σ_θ	s
Gamma	Subscale	500	0.143	0.396	0.236	0.108	0.131	0.295	0.167	0.087
		1000	0.102	0.244	0.159	0.094	0.091	0.171	0.089	0.080
		2000	0.077	0.103	0.039	0.092	0.069	0.086	0.032	0.079
	Bifactor	500	0.140	0.459	0.241	0.110	0.127	0.347	0.228	0.088
		1000	0.094	0.326	0.196	0.102	0.084	0.250	0.186	0.085
		2000	0.073	0.245	0.177	0.093	0.066	0.196	0.161	0.072

Table 10: RMSE and aBias for the Gamma Model within the Main-Effect HO-GRCDM

D.2 Additional Simulation for the Gamma Model within a Main-Effect CDM

Like the other simulations conducted for main-effect models, we also set the coefficients β_k^j according to:

$$\beta_0^j = c_0, \quad \beta_k^j = \frac{c_1}{\sum_{k=1}^K q_{jk}}, \quad \forall j \in [J], k \in [K],$$

where (c_0, c_1) are two constants, set to (1, 2) for the Gamma-CDMs. These constants are chosen to match the scale of parameters obtained in the Empirical Data Analysis section. The estimation procedure is shown in Algorithm 1 in the paper. Here, the built-in R function *optim* is used to estimate the shape parameters. The obtained RMSE and aBias are presented in Table 10, and the proportion of correctly recovered rows and entries are shown in Table 11. It can be seen that the estimation accuracy of both model parameters and Q improves as the sample size grows.

	N	500	1000	2000
Subscale	P_R	0.727	0.787	0.854
	P_E	0.954	0.965	0.978
Bifactor	P_R	0.726	0.778	0.851
	P_E	0.955	0.965	0.977

Table 11: Proportion of Correctly Recovered Rows (P_R) and Entries (P_E) in Q-matrix for the Gamma Model within the Main-Effect HO-GRCDM

E Additional Simulation Results under a Fully Exploratory Setting

In this appendix, we conduct an additional simulation study under a fully exploratory setting, where no prior information is available for either the bottom-layer Q-matrix or the higher-layer $Q^{(H)}$-matrix. Similar to the simulations presented in the main text, we consider three sample sizes: $N = 500, 1000$, and 2000, under the configuration $(J, K, D) = (30, 7, 3)$. To keep the scope focused, we present a representative simulation under the main-effect model for the bottom layer and a subscale structure for the higher layer. For the bottom-layer model, we consider three types of response models: (a) Bernoulli CDM for binary data, (b) Poisson CDM for count data, and (c) Lognormal CDM for continuous data. The bottom-layer Q-matrix and the higher-order true slope coefficients are set identically to $Q_{30\times 7}$ and $\boldsymbol{\lambda}_1^{\text{subscale}}$ in Equation (45) of the main text. The model remains identifiable, as $Q_{30\times 7}$ and $\boldsymbol{\lambda}_1^{\text{subscale}}$ satisfy the conditions in Theorem 2 of Section 5. In this simulation, we use known values of K and D to focus on evaluating the estimation performance of the fully exploratory HO-GRCDM, without the added complexity of model selection. Other simulation settings, including the Monte Carlo sample size, convergence criterion, and initialization, remain consistent with those in the Simulation Study in Section 6.

After the algorithm terminates, the Hungarian algorithm (Kuhn, 1955) is applied twice to align the estimated coefficient matrices with the true ones. First, it is used to reorder the columns of the bottom-layer coefficient matrix and, accordingly, the rows of the higher-layer coefficient matrix. Second, it is applied to reorder the columns of the higher-layer coefficient matrix. Next, RMSE and aBias are computed to assess estimation accuracy. The results, presented in Table 12, show that estimation errors remain reasonably small, and the accuracy of all parameters improves as the sample size increases, validating model identifiability and estimation consistency. The performance in parameter estimation is generally consistent with the partially exploratory scenario for the bottom layer but is worse for the higher layer. This is expected due to the inherent challenges of recovering two latent layers in an exploratory setting.

Model	N	RMSE				aBias			
		β	$\boldsymbol{\lambda}$	$\boldsymbol{\Sigma}_\theta$	γ	β	$\boldsymbol{\lambda}$	$\boldsymbol{\Sigma}_\theta$	γ
Lognormal	500	0.172	0.210	0.103	0.031	0.153	0.164	0.081	0.025
	1000	0.136	0.185	0.086	0.023	0.125	0.151	0.067	0.019
	2000	0.114	0.165	0.072	0.016	0.107	0.140	0.058	0.012
Poisson	500	0.163	0.469	0.196	–	0.132	0.415	0.173	–
	1000	0.117	0.414	0.181	–	0.098	0.377	0.159	–
	2000	0.086	0.355	0.151	–	0.075	0.332	0.133	–
Bernoulli	500	0.376	0.237	0.108	–	0.327	0.194	0.087	–
	1000	0.321	0.206	0.087	–	0.287	0.176	0.071	–
	2000	0.274	0.189	0.058	–	0.251	0.171	0.048	–

Table 12: RMSE and aBias for the Main-Effect HO-GRCDM under a fully exploratory setting.

F Additional Details for the TIMSS Data

Table 13: Descriptive item information. In the second column, MC denotes multiple choice items and CR denotes constructed response items.

Item	Item Type	Label	Slope	Location
1	MC	Octagon with equivalent shading	1.65	0.55
2	CR	Time when Pat finishes last lap	1.28	-0.26
3	MC	Multiples of 3	1.20	0.68
4	CR	Convert decimal to a fraction	1.24	0.39
5	MC	Expression for area of rectangle	1.32	0.67
6	MC	Expression with exponents of y	1.01	0.11
7	CR	Number of matches for figure 10	0.86	0.29
8	MC	Graph of y = 2x	1.24	1.62
9	MC	Rotation and reflection	1.13	1.59
10	MC	Surface area of the prism	1.50	0.99
11	MC	Value of angle x outside triangle	1.20	0.27
12	MC	Number of balls in a bag	1.19	-0.10
13	MC	Liv's smartphone use	1.91	0.76
14	CR	Statements for all values of integer a	0.74	1.04
15	MC	Arrow to show 5/12 on number line	1.49	0.74
16	CR	Value of fraction X in square	1.32	1.13
17	CR	Number of blue beads on bracelet	0.74	0.07
18	MC	Value of 2(6x - 3y)	1.29	0.09
19	MC	Expression equivalent to 2y + 6xy2	0.86	0.66
20	CR	Formula for stopping distance	1.16	0.66
21	CR	Value of x given perimeter of triangle ABC	1.60	0.92
22	MC	Additional point on a straight line	1.25	0.75
23	CR	Value of angle x in a quadrilateral	1.31	-0.15
24	CR	Methods of folding paper	0.50	0.33
25	CR	Coordinates to complete KLMN	1.23	0.72
26	CR	Mean temperature for 5 days	1.51	0.69
27	CR	Best graph for town information	1.57	0.14
28	CR	Bar graph of newspaper sales	1.05	1.47

Note: The Slope and Location refer to the item slope and location parameters obtained by fitting an item response model.

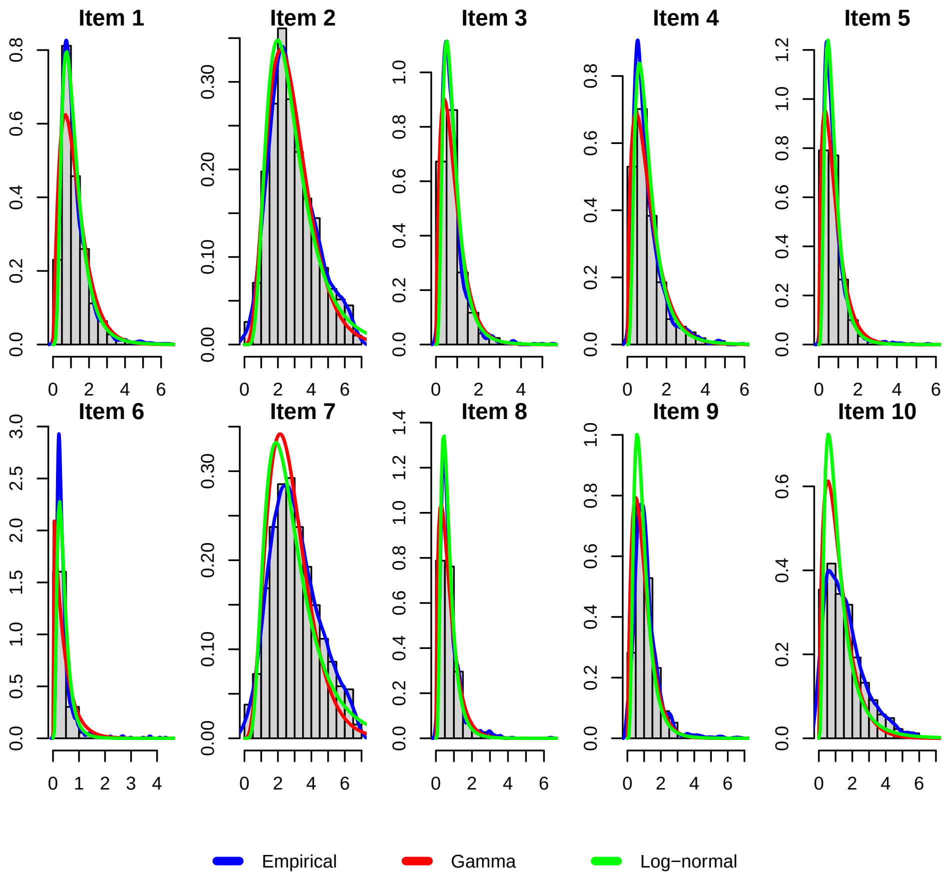

Figure 3: Probability Histogram and Fitted Density Curves (Empirical Density, Gamma Model, and Log-Normal Model) for Response Time Data (in Minutes) for Items 1-10.

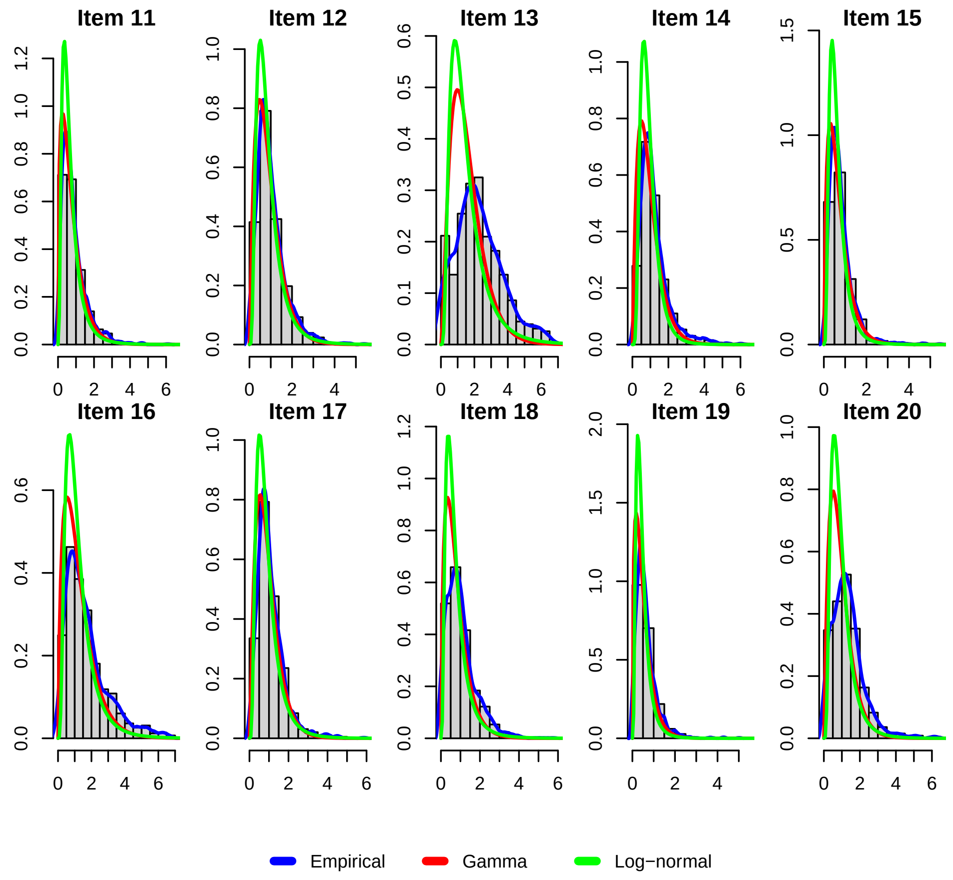

Figure 4: Probability Histogram and Fitted Density Curves (Empirical Density, Gamma Model, and Log-Normal Model) for Response Time Data (in Minutes) for Items 11-20.

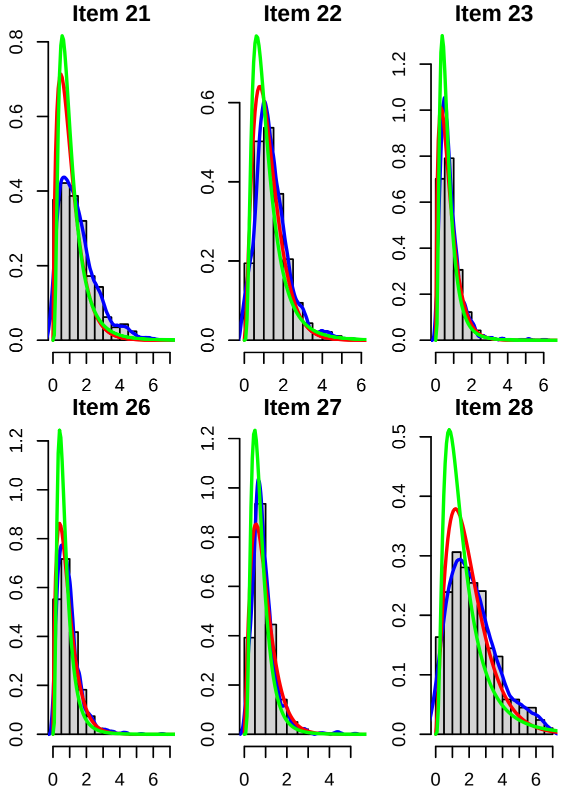

Figure 5: Probability Histogram and Fitted Density Curves (Empirical Density, Gamma Model, and Log-Normal Model) for Response Time Data (in Minutes) for Items 21-28.

References

Anderson, T. and Rubin, H. (1956). Statistical inference in. In *Proceedings of the Berkeley Symposium on Mathematical Statistics and Probability*, page 111. University of California Press.

Bishop, C. M. and Nasrabadi, N. M. (2006). *Pattern recognition and machine learning*, volume 4. Springer.

Chatterjee, S. (2015). Matrix estimation by universal singular value thresholding. *Annals of Statistics*, pages 177–214.

Chen, Y., Liu, J., Xu, G., and Ying, Z. (2015). Statistical analysis of Q-matrix based diagnostic classification models. *Journal of the American Statistical Association*, 110(510):850–866.

Fang, G., Guo, J., Xu, X., Ying, Z., and Zhang, S. (2021). Identifiability of bifactor models. *Statistica Sinica*, 31:2309–2330.

Gelman, A., Carlin, J. B., Stern, H. S., Dunson, D. B., Vehtari, A., and Rubin, D. B. (2013). *Bayesian Data Analysis*. CreateSpace.

Kuhn, H. W. (1955). The hungarian method for the assignment problem. *Naval research logistics quarterly*, 2(1-2):83–97.

Lee, S. and Gu, Y. (2025). Deep discrete encoders: Identifiable deep generative models for rich data with discrete latent layers. *arXiv preprint arXiv:2501.01414*.

Li, J., Gibbons, R., and ková, V. R. (2025). Sparse Bayesian multidimensional item response theory. *Journal of the American Statistical Association*, (just-accepted):1–32.

Zhang, H., Chen, Y., and Li, X. (2020). A note on exploratory item factor analysis by singular value decomposition. *Psychometrika*, 85(2):358–372.
